# Not All Extracellular Vesicles Are Equal: A Narrative Comparative Review of Source-Dependent Function and Therapeutic Potential

**DOI:** 10.3390/cells15191731

**Published:** 2026-09-23

**Authors:** Yasaman Zandi Mehran, Michael Weber, Fateme Hoseinzade

**Affiliations:** 1 Biomedical Engineering, Regenerative Medicine Equipment, Dubai 25048, United Arab Emirates; 2 European Laser Academy, 45657 Recklinghausen, Germany; praxisdrmweber@gmx.com; 3 Physical Medicine and Rehabilitation, Isfahan University of Medical Sciences, Isfahan 81746-73461, Iran; fatemehoseinzade98@gmail.com

**Keywords:** cancer immunotherapy, drug delivery, extracellular vesicles, mesenchymal stromal cells, regenerative medicine

## Abstract

Extracellular vesicles (EVs) are increasingly investigated as cell-free therapeutic platforms, yet EVs from different cellular sources are often considered functionally interchangeable despite substantial biological heterogeneity. This narrative comparative review evaluated how EV source influences molecular cargo, phenotype, biological function, therapeutic suitability, safety, and translational potential. PubMed/MEDLINE, Europe PMC, and OpenAlex were searched from inception through 14 August 2026, supplemented by targeted Google Scholar and backward and forward citation searching. Of the 9779 database records identified, 3799 duplicates were removed and 5980 unique records were screened. Sixty-seven database studies and three additional studies identified through supplementary searching met the criteria for direct within-study comparison of EVs from distinct parental cell or tissue sources. Across the 70 direct comparisons, source-associated differences in cargo, phenotype, target-cell responses, and therapeutic performance were frequently reported; however, the depth and consistency of supporting evidence varied substantially across source families and indications, with activity further influenced by cellular state, engineering, vesicle fraction, isolation method, and dose. Based on these findings, we propose EV-FIT+—Function, Indication, Trade-off, and Contra-Function—as an author-developed qualitative framework for rational EV source selection. No EV source is universally superior; therapeutic suitability depends on matching source- and product-specific biological characteristics and risks to the intended clinical objective.

## 1. Introduction

Extracellular vesicles are lipid-bilayer-delimited particles released by cells into the extracellular environment and capable of transferring proteins, lipids, nucleic acids, metabolites, receptors, and signaling components between cells. MISEV2023 emphasizes the use of the generic term extracellular vesicle when the precise subcellular origin has not been demonstrated and recommends transparent reporting of EV preparation, characterization, and functional studies [1].

Interest in EV therapeutics has grown rapidly because vesicles may reproduce selected paracrine functions of living cells while offering potential advantages for cell-free administration, storage, engineering, and standardization. The expansion of the field, however, has encouraged an oversimplified concept in which ‘exosome therapy’ is sometimes treated as a single modality. Biologically, such a concept is difficult to justify.

An NK cell specializes in the recognition and destruction of abnormal cells. A cytotoxic T lymphocyte uses a distinct adaptive recognition program. A dendritic cell specializes in antigen processing and immune instruction. A neural stem/progenitor cell supports neural repair programs, whereas a cardiomyocyte, placental-MSC, Wharton’s jelly MSC, adipose MSC, and bone-marrow MSC each possess different transcriptional, proteomic, metabolic, and signaling states. There is therefore little reason to expect vesicles produced by these cells to be functionally equivalent.

Direct experiments support this source dependency. Vδ2 γδ-T-cell-derived EVs demonstrated a combination of direct tumor-cell cytotoxicity and adaptive immune stimulation in an Epstein–Barr virus-associated tumor model [2]. Direct wound-healing comparisons of BM-, adipose-, and UC-MSC-derived EVs showed that relative effects depended on both the source and recipient cell [3]. Proteomic comparisons likewise revealed source-specific molecular programs among these MSC-derived preparations [4].

Accordingly, this review examines how EV source and cellular state relate to biological function, therapeutic suitability, and relevant biological and manufacturing trade-offs across indications.

### Nomenclature and Conceptual Scope

Exosome is not synonymous with extracellular vesicle. When endosomal biogenesis has not been demonstrated, operational terms such as EV or small extracellular vesicle (sEV) are generally more defensible [1]. Throughout the narrative text, EV or sEV terminology is therefore used unless endosomal biogenesis has been demonstrated. When an original study or clinical product specifically used the term “exosome,” that designation is retained only where necessary to accurately describe the source report; published article titles remain unchanged in the reference list. To avoid conflating different meanings of “source,” three source descriptors are distinguished throughout this review. Cell source refers to the immediate EV-producing cell type or lineage, such as NK cells, dendritic cells, MSCs, neurons, or cardiomyocytes. Tissue source refers to the anatomical tissue or compartment from which the EV-producing cells or, where applicable, the EV preparation itself originated, such as bone marrow, adipose tissue, umbilical cord/Wharton’s jelly, or placenta. Engineered source refers specifically to a parental-cell population deliberately modified before EV production, such as CAR-T or CAR-NK cells. Engineering performed after EV isolation, including exogenous cargo loading or surface modification, was considered product engineering rather than source engineering. These descriptors are not mutually exclusive: for example, BM-MSC EVs have an MSC cell source and bone-marrow tissue origin, whereas CAR-T EVs have a T-cell source with engineered parental-cell status.

This distinction is especially important in source comparisons because purification methods can enrich different vesicle populations and co-isolates. Apparent source-dependent effects can therefore be confounded by isolation, size fraction, purity, media composition, and dose normalization.

We therefore resolve source identity into three complementary descriptors within the product-identity hierarchy: [Cell Source + Tissue/Anatomic Origin + Parental-Cell Engineering Status] → Donor/Cell State → Culture and Microenvironmental Context → Vesicle Population → Manufacturing → Cargo → Potency → Risk. A label such as “NK-cell EV” specifies a cell lineage but not necessarily tissue origin or cellular state, whereas “umbilical-cord EV” specifies an anatomical origin but may not identify the EV-producing cell type. Neither designation alone is sufficient for therapeutic product definition unless the relevant source dimensions, cell state, vesicle fraction, manufacturing process, and mechanism-linked potency assay are also specified.

## 2. Materials and Methods

### 2.1. Review Objective

The primary objective is to determine how EV-producing cell identity, tissue/anatomic origin, and parental-cell engineering status influence molecular cargo, surface phenotype, biological function, therapeutic suitability, safety, engineering potential, scalability, manufacturing reproducibility, and translational development stage. For this review, therapeutic suitability was operationally defined as the qualitative degree of fit between a specific EV source/product and an intended therapeutic indication. This fit was interpreted according to: (i) alignment between the EV’s demonstrated biological function and the required therapeutic function; (ii) mechanistic and potency support; (iii) indication-specific trade-offs, contra-functions, and safety risks; (iv) manufacturing feasibility, reproducibility, and scalability; (v) the strength and stage of supporting translational evidence. Therapeutic suitability was not considered synonymous with demonstrated clinical efficacy, therapeutic readiness, or regulatory approval. Direct head-to-head source comparisons receive the greatest interpretive weight because indirect comparisons across laboratories are strongly confounded by methods and disease models.

### 2.2. Search Strategy

A reproducible literature search was performed in PubMed/MEDLINE, Europe PMC, and OpenAlex from database inception through 14 August 2026. Search blocks combined EV terms with source terms and comparative terms; the exact database-specific strategies and raw identification counts are provided in Supplementary Table S1. The final searches identified 5527 records in PubMed/MEDLINE, 3633 in Europe PMC, and 619 in OpenAlex. Google Scholar was used only for targeted supplementary searching, together with backward and forward citation searching of key comparative studies; approximate Google Scholar hit counts were not treated as database identification counts. Subscription-only Embase, Scopus, and Web of Science Core Collection were not included because reproducible complete exports were not available for this review. This restriction is reported explicitly as a review limitation.

### 2.3. Eligibility Criteria and Data Extraction

The primary search layer included original peer-reviewed mammalian studies directly comparing EV preparations from at least two distinct parental cell or tissue sources within the same study. For this review, a direct head-to-head comparison was defined as one in which EVs from the different sources were prepared using the same or methodologically comparable isolation/purification approach, evaluated under the same or substantially matched experimental conditions, and compared using at least one common outcome assessed by the same method. Studies in which major differences in EV isolation, experimental model, treatment conditions, or outcome assessment prevented attribution of the observed differences to EV source were not classified as direct head-to-head comparisons. Eligible outcomes included source-dependent cargo or surface phenotype, uptake, mechanism, potency, therapeutic function, safety or contra-function, yield/manufacturing characteristics, and translational performance. Reviews, editorials, protocols, conference-only records, preprints, biomarker-only studies, single-source EV studies, EV-versus-parent cell comparisons without at least two EV sources, same-source preconditioning-only comparisons, isolation-method-only comparisons, and non-mammalian or microbial vesicle studies were excluded from the primary search layer (Table 1). Source-specific mechanistic or clinical studies that did not meet the direct-comparison criterion were retained only as contextual narrative evidence. These contextual studies were not counted among the 70 studies in the primary direct-comparison layer and were not treated as equivalent to within-study comparative evidence; they were used only to support interpretation of biological mechanisms, safety, manufacturing considerations, and translational relevance.

For each direct-comparison study, we extracted the publication year, EV cellular/tissue sources, study context, comparative endpoint, DOI/PMID, and the direction or nature of the source-dependent result where reported. For major comparative conclusions, we also considered the number of direct-comparison studies contributing to that conclusion and whether their findings were broadly concordant, mixed or context-dependent, or materially discordant. These patterns were interpreted in relation to the similarity of the source comparison, experimental model, target cell, endpoint, dose, isolation approach, and vesicle fraction rather than by simple numerical vote counting. For qualitative evidence mapping, we additionally recorded the directness and comparability of source comparisons, evidence of independent replication, the presence of in vivo or advanced translational confirmation, reported cargo–mechanism relationships, experimental evidence supporting those relationships, and the highest documented translational stage. Evidence was then mapped to cargo/phenotype, functional potency, therapeutic fit as operationally defined in Section 2.1, safety trade-offs, and translational development stage. The review distinguishes this direct-comparison layer from broader source-specific mechanistic and translational evidence used to interpret biological plausibility and clinical relevance. Safety findings were interpreted at the level of the evaluated EV preparation rather than automatically attributed to the entire parental-cell source. Where reported, interpretation considered the EV fraction or size range, isolation/manufacturing approach, administered dose, route of administration, and other relevant exposure conditions.

### 2.4. Deduplication, Screening, and Synthesis

The three database exports contained 9779 records. Records were merged and deduplicated using DOI and PMID identifiers supplemented by normalized exact-title matching, removing 3799 duplicate records and leaving 5980 unique records for title/abstract screening. Screening applied the predefined direct source-comparison criteria described in Section 2.3, with computer-assisted keyword prioritization and manual eligibility verification of retained candidate records. Sixty-seven database records met the strict within-study source-comparison criteria. Targeted Google Scholar and backward/forward citation searching identified three additional unique eligible studies, yielding 70 direct source-comparison studies in the search layer. Accordingly, the numerical study count and the primary study inventory refer exclusively to this direct-comparison layer; broader mechanistic, translational, or clinical studies used for contextual interpretation were not included in the n = 70 count. Because study designs, source pairs, dose metrics, model systems, and outcomes were highly heterogeneous, no meta-analysis was attempted. Findings were synthesized narratively by functional and source category, with descriptive study counts used to indicate the depth of direct-comparison evidence supporting major conclusions. Findings were considered broadly concordant when sufficiently comparable studies supported the same general interpretation, mixed or context-dependent when relative performance varied according to model, target cell, endpoint, dose, or experimental conditions, and materially discordant when sufficiently comparable studies yielded opposing conclusions. These study counts were treated as descriptive indicators of evidence depth rather than statistical weights or pooled estimates of effect. The review was not prospectively registered and no separate protocol was published. A single-reviewer workflow was used for screening and structured extraction. Given the heterogeneous preclinical, mechanistic, and translational study designs, no single validated risk-of-bias instrument was imposed across all studies; instead, study-level methodological reporting domains are summarized in Supplementary Table S5 and are not presented as a validated risk-of-bias score. Deduplication and bibliographic organization were performed with custom Python 3 scripts using pandas; title/abstract screening prioritization used predefined source and comparison terms.

### 2.5. Evidence Layers

For comparative interpretation, the included evidence was organized into four tiers according to the directness of source comparison and the type of evidence provided (Table 2). Tier I included direct head-to-head studies meeting the criteria defined in Section 2.3: EVs from two or more distinct parental cell or tissue sources were compared within the same study using the same or methodologically comparable isolation/purification approach, substantially matched experimental conditions, and at least one common outcome assessment. These studies received the greatest interpretive weight. Tier II comprised disease-matched evidence in which different EV sources were evaluated in similar disease contexts but not within the same experimental framework. Tier III included mechanistic evidence addressing EV cargo, receptors, signaling pathways, recipient-cell effects, or causal mechanisms. Tier IV comprised translational evidence, including large-animal studies, human administration, clinical trials, and studies addressing GMP-compatible or scalable manufacturing. This hierarchy was used to structure interpretation of the evidence and was not intended as a formal risk-of-bias or certainty-of-evidence grading system. The Tier I–IV hierarchy describes the type and directness of evidence and is conceptually distinct from the source-level CEC, TDS, and CMEC descriptors described below. Within this hierarchy, Tier I represents the primary direct-comparison evidence layer, whereas Tiers II–IV provide complementary contextual evidence for disease-specific interpretation, mechanistic plausibility, and translational relevance and were not considered substitutes for Tier I comparisons.

### 2.6. Qualitative Evidence-Mapping Criteria

To distinguish comparative confidence, translational development stage, and mechanistic support, three author-developed qualitative evidence-mapping descriptors were applied at the EV-source level: Comparative Evidence Category (CEC), Translational Development Stage (TDS), and Cargo–Mechanism Evidence Category (CMEC). These descriptors were developed for descriptive evidence mapping in the present narrative review and should not be interpreted as validated quantitative scoring systems, risk-of-bias assessments, certainty-of-evidence grades, regulatory-readiness measures, or clinical decision instruments. Their conceptual basis was informed by recommendations emphasizing rigorous EV characterization, appropriately controlled functional studies, and mechanism-relevant assessment of therapeutic EV activity [1,5]. CEC, TDS, and CMEC were intentionally evaluated separately because the strength of comparative evidence, progression toward clinical translation, and mechanistic confidence represent different dimensions of the evidence base. These three descriptors were not prospectively predefined before the literature search and were not used to determine study eligibility. Rather, they were developed during interpretive synthesis after assembly of the primary direct-comparison evidence layer, and their operational criteria were subsequently formalized and applied consistently across EV-source categories.

CEC reflected the directness, comparability, replication, and biological depth of evidence comparing EV sources. CEC-A (strong comparative evidence) required either (i) at least two independent substantially matched head-to-head source comparisons with generally concordant findings or (ii) a substantially matched head-to-head comparison supported by both in vivo confirmation and mechanistic evidence. CEC-B (moderate comparative evidence) required at least one direct within-study source comparison but lacked the replication or combined in vivo and mechanistic support required for CEC-A; multiple concordant disease-matched comparative studies could also support this category. CEC-C (source-specific evidence) indicated evidence of biological activity from multiple studies but an absence of robust standardized source-to-source comparisons. CEC-D (emerging evidence) indicated predominantly limited, unreplicated proof-of-concept or engineered-platform evidence. In applying CEC, the number of relevant comparisons was considered together with methodological comparability and consistency of findings; a larger number of studies did not automatically strengthen a superiority claim when the underlying comparisons were heterogeneous or their findings were context-dependent or materially discordant.

TDS was used to describe the highest documented stage of translational development reached by each EV-source category rather than overall therapeutic readiness. TDS-0 indicated mechanistic or in vitro evidence only; TDS-1, therapeutic evidence in small-animal models; TDS-2, large-animal or advanced translational-model evidence; TDS-3, human administration or Phase I therapeutic evaluation; and TDS-4, Phase II or later therapeutic evaluation [6]. The highest clearly documented stage was assigned irrespective of the volume of evidence at earlier stages. Importantly, advancement to human administration or a higher clinical development stage was not considered sufficient to establish therapeutic readiness. Therapeutic readiness was interpreted separately and qualitatively, where evidence was available, by considering manufacturing reproducibility and scalability, mechanism-linked potency, product safety, biodistribution, batch-to-batch consistency, and evidence of therapeutic efficacy [1,5]. For clinical interpretation, developmental stage, therapeutic efficacy, and comparative clinical evidence were considered separately. Translational/clinical development stage refers to the highest level of development reached, including large-animal evaluation, human administration, or clinical-trial phase. Therapeutic efficacy refers specifically to evidence that an EV intervention produces clinically meaningful benefit in treated patients. Comparative clinical evidence requires a direct clinical comparison between EV products or source categories. Accordingly, large-animal studies, GMP production, human administration, or progression to Phase I/II were not by themselves considered evidence of therapeutic efficacy or comparative clinical superiority.

CMEC reflected the strength of evidence linking identified EV cargo to a biological mechanism and demonstrated function. CMEC-0 indicated that no specific cargo–mechanism relationship had been demonstrated. CMEC-1 indicated descriptive or associative evidence only, such as detection or enrichment of a candidate cargo, correlation with a biological phenotype, or pathway association, without experimental perturbation demonstrating functional involvement. CMEC-2 indicated mechanistic support in which manipulation or inhibition of the candidate cargo, pathway, or relevant receptor altered the EV-mediated phenotype, but cargo-specific causality remained incomplete. CMEC-3 indicated strong causal evidence in which direct manipulation of the implicated cargo produced a corresponding change in EV-mediated function and was supported by additional confirmation, such as verification of altered cargo abundance in EVs, rescue experiments, reciprocal gain-/loss-of-function approaches, receptor/pathway validation, or confirmation in an independent experimental model.

For CEC and CMEC, the highest category whose criteria were fully satisfied was assigned; when evidence was insufficient to distinguish two adjacent categories, the lower category was assigned conservatively. TDS was assigned according to the highest clearly documented translational stage and was not interpreted as a composite measure of therapeutic readiness. These classifications were applied to source categories as qualitative evidence-mapping descriptors rather than as study-level quality scores. Following definition of the operational criteria, source-level CEC, TDS, and CMEC assignments were independently rechecked by two authors, with discrepancies resolved by consensus.

## 3. Results

The formal database searches identified 9779 records (PubMed/MEDLINE, n = 5527; Europe PMC, n = 3633; OpenAlex, n = 619). After 3799 duplicates were removed, 5980 unique records underwent title/abstract screening and 5913 were excluded for not meeting the strict direct source-to-source EV comparison criteria. Sixty-seven database reports were retained for eligibility verification and inclusion. Targeted Google Scholar, backward reference screening, and forward citation searching identified three additional unique eligible reports. The final search layer therefore comprised 70 direct source-comparison studies.

### 3.1. Characteristics of the Included Direct Comparisons

The 70 included direct-comparison studies span immune, stromal/regenerative, perinatal, neural, cardiovascular/pluripotent, epithelial, platelet, and other tissue-specialized EV sources, although the evidence base remains dominated by comparisons among mesenchymal stromal cell sources. This concentration was substantial: 52 of the 70 direct-comparison studies included at least one MSC source, whereas matched comparisons involving several immune-effector, neural, cardiac, and engineered EV platforms were much less numerous. The numerical depth of evidence therefore differed markedly among source families. Across these studies, source-dependent differences were reported in molecular cargo, surface phenotype, yield, recipient-cell response, and disease-model efficacy. Representative comparisons include Vδ2-T versus NK/DC EVs in tumor control, BM/AD/UC-MSC EV comparisons in wound healing and proteomics, six-source cardiac-repair comparisons, and more recent comparisons involving immune-cell subsets, brain endothelial cells, platelets, epithelial cells, iPSC-derived products, neural-lineage cells, and tissue-specialized EVs [2,3,4,6,7,8,9,10,11,12,13,14,15,16,17,18,19,20,21,22,23,24,25,26,27,28,29,30,31,32,33]. The full included-study inventory is provided in Supplementary Table S4. These 70 studies constitute the primary direct-comparison evidence layer; source-specific mechanistic, translational, and clinical studies discussed subsequently are used as contextual evidence and are not part of this numerical study set. Collectively, these comparisons frequently identified source-associated differences in EV phenotype and performance, but the number and reproducibility of studies supporting specific source rankings varied substantially. Observed effects were additionally modified by cellular state, vesicle fraction, manufacturing, dose, and indication.

### 3.2. A Function-Based Taxonomy of Therapeutic EV Sources

EVs should be classified not only according to size or parent-cell name but also according to the dominant biological task their source is equipped to perform (Figure 1). The categories below deliberately overlap because EV function exists on a spectrum rather than in rigid boxes (Table 3). Because this taxonomy is function-based, the “representative sources” include both native cell lineages and engineered parental-cell variants. CAR-T and CAR-NK are therefore treated as engineered forms of T-cell and NK-cell sources, respectively, rather than as independent tissue-source categories. Likewise, BM-, AD-, and UC/WJ-MSC labels combine a shared MSC cell identity with distinct anatomical tissue origins.

### 3.3. Source → Cargo → Mechanism → Function

The therapeutically relevant content of EVs should be distinguished from generic identity markers. CD9, CD63, CD81, ALIX, or TSG101 may support characterization but do not explain why a preparation kills a tumor cell, suppresses inflammation, protects a neuron, or promotes angiogenesis. Importantly, identification or enrichment of a cargo molecule does not by itself establish that the cargo mediates the observed biological effect. Cargo–function relationships may range from descriptive association to experimentally supported causality. Accordingly, this review distinguishes cargo detection or association from mechanistic evidence based on perturbation, direct manipulation, rescue, or other confirmatory experiments, as reflected in the CMEC framework described in Section 2.6. These source-dependent relationships between EV origin, molecular cargo, biological mechanism, and functional outcome are summarized in Figure 2.

### 3.4. Immune-Effector and Immune-Regulatory EVs

NK-derived EVs are attractive for applications requiring direct cytotoxic activity. Human NK EVs can contain perforin, granzymes, granulysin, FasL, and other immune-associated molecules, and experimental studies demonstrate multiple mechanisms of cancer-cell killing [34].

Their major advantage is biologically coherent: the parent cell is naturally optimized to destroy stressed, infected, or malignant targets. However, NK-EV potency is strongly state-dependent. Cytokine or hypoxic conditioning can alter EV yield and cytotoxic phenotype, and memory-like NK-derived sEVs represent a distinct product from resting primary NK or NK-cell-line EVs [41].

Disadvantages include comparatively limited human therapeutic evidence, complex large-scale expansion, variability between primary NK cells and immortalized lines, incomplete understanding of off-target cytotoxicity, and the absence of a universally accepted mechanism-linked potency assay. A meaningful potency assay for NK-EVs should measure functional tumor killing rather than particle number alone.

### 3.5. Cytotoxic T-Cell and γδ-T-Cell EVs

The label ‘T-cell EV’ is too broad to predict therapeutic behavior. EVs from CD8 cytotoxic T lymphocytes, γδ-T cells, Treg cells, exhausted T cells, and engineered CAR-T cells have different biological programs. The available matched evidence for the specific comparison of Vδ2-T, NK-, and dendritic-cell EVs currently derives from a single direct study. In an EBV-associated tumor model, Vδ2-T EVs combined direct cytotoxicity with antigen-associated immune activation and performed favorably across the tested cytotoxic and immune-activation endpoints [2]. Because this comparative ranking has not been independently replicated in the same experimental setting, it should be interpreted as model- and endpoint-specific rather than as evidence of general Vδ2-T superiority. Potential disadvantages of γδ-T EVs include limited standardized manufacturing, donor variability, relatively few head-to-head studies outside selected tumor systems, and very limited human EV-therapy evidence.

### 3.6. CAR-T and CAR-NK EVs

CAR-T and CAR-NK are considered engineered parental-cell sources in this review: their underlying cellular identities remain T cell and NK cell, respectively, while the CAR modification constitutes an additional engineering descriptor. CAR-T cells can release EV preparations reported as exosomes that display CAR molecules while retaining cytotoxic cargo such as perforin and granzyme B. These vesicles can produce antigen-dependent tumor-cell killing in preclinical models [35]. Anti-HER2 CAR-NK92-derived EVs further support the concept that CAR engineering can be transferred into an NK-vesicle platform [42].

The principal advantage is programmable specificity. The major trade-offs are antigen escape, target heterogeneity, manufacturing complexity, heterogeneity in CAR incorporation among vesicles, the need to quantify functional receptor density, and predominantly preclinical evidence. CAR engineering therefore provides programmable antigen specificity while retaining the associated limitations of target heterogeneity and antigen escape.

### 3.7. Dendritic-Cell-Derived EVs

Dendritic-cell-derived EVs occupy a different therapeutic niche. Their principal strength is immune instruction through peptide–MHC and costimulatory biology rather than maximal direct cytotoxicity. Antigen-bearing dendritic-cell EVs can support antigen-specific T-cell activation [36].

DC-EVs have one of the longest clinical development histories among EV platforms. A Phase I metastatic-melanoma study established the feasibility of GMP-scale production and administration, while a later Phase II NSCLC study demonstrated immunological activity through stimulation of the NK-cell arm of antitumor immunity [43,44]. These findings support a relatively advanced clinical development stage and provide manufacturing, feasibility, and safety experience. However, clinical development stage should be distinguished from therapeutic efficacy: these studies do not by themselves establish clinically meaningful therapeutic benefit. They also do not provide comparative clinical evidence of superiority over NK, CAR, or other EV platforms.

Trade-offs include weaker direct killing, antigen/HLA dependence, maturation-state dependence, and manufacturing complexity in autologous strategies.

### 3.8. Regulatory T-Cell-Derived EVs

Treg-derived EVs illustrate the opposite end of the immune spectrum (Figure 3). The EV-mediated transfer of Let-7d from Treg cells can suppress pathogenic Th1 responses, while the Treg EV transfer of miR-150-5p and miR-142-3p can push dendritic cells toward a more tolerogenic phenotype [37,38].

These properties make Treg EVs attractive for inflammatory, autoimmune, or transplant-tolerance applications, but the same immunosuppressive program could be undesirable in cancer or persistent infection. Accordingly, their immunosuppressive activity requires indication-specific evaluation (Figure 4).

### 3.9. Regenerative and Perinatal EVs

#### 3.9.1. Umbilical Cord/Wharton’s Jelly MSC EVs

UC/WJ-MSC EVs are attractive because the parent cells are relatively young, expandable, and accessible from perinatal tissue. Their reported functions include immunomodulation, angiogenesis, antiapoptotic signaling, and tissue repair. A mechanistically defined example is EV-associated miR-181c, which reduced burn-associated inflammation through suppression of TLR4 signaling [39].

Clinical translation is emerging: a 2024 first-in-human Phase I study evaluated intrathecal allogeneic UC-MSC-derived exosomes in subacute spinal cord injury and reported an acceptable safety profile in the treated cohort [45]. Accordingly, this first-in-human experience represents early clinical development and provides preliminary safety information, but it should not be interpreted as evidence of established therapeutic efficacy. Because the study was an early single-arm evaluation, it also does not provide comparative clinical evidence between EV sources. Controlled efficacy data, together with reproducible manufacturing, potency, biodistribution, batch consistency, and longer-term safety, remain necessary for further translation.

Disadvantages are equally important. Source and manufacturing variability can alter potency. Regenerative, immunosuppressive, or angiogenic signaling may be undesirable in selected cancers. Large UC-MSC-derived EV fractions can display tissue-factor-dependent procoagulant activity; high intravenous doses caused pulmonary thromboembolism in mice in a dose- and TF-dependent manner [46]. This finding applies to the studied large-EV preparation and should not be generalized automatically to every UC-MSC sEV product, but it demonstrates why vesicle fraction, isolation/manufacturing method, TF/CD142 activity, dose, route, and infusion conditions must be incorporated into product-specific safety evaluation.

#### 3.9.2. Placental MSC EVs

Placental-MSC-derived EVs have demonstrated proangiogenic activity in vitro and in vivo, including enhanced endothelial migration, tube formation, angiogenesis-related gene expression, and blood flow in an ischemic model [47].

Potential advantages include strong immunoregulatory and regenerative biology and access to perinatal tissue. Disadvantages include heterogeneity across placental compartments, donor and gestational context, incomplete definition of the dominant active cargo, limited direct comparisons with WJ/UC sources, and concern that proangiogenic or immunosuppressive functions may be inappropriate in selected oncological contexts.

#### 3.9.3. Bone Marrow, Adipose, and UC-MSC Comparisons

A direct wound-healing comparison under serum- and xeno-free conditions showed that BM-, adipose-, and UC-MSC EVs all enhanced repair-associated behavior but did not produce identical outcomes. BM-MSC EVs produced particularly strong fibroblast responses, whereas UC-MSC preparations showed strong keratinocyte migration effects [3].

Proteomic analysis of these three MSC tissue-of-origin groups also demonstrated source-dependent molecular signatures, providing a mechanistic basis for functional divergence [4]. A 2025 osteoarthritis comparison further reported stronger effects of BMSC- and umbilical MSC-derived EVs than adipose-derived preparations across several inflammatory and cartilage-related endpoints in that experimental framework [7].

The broader direct-comparison inventory provides substantially greater replication for MSC tissue-of-origin comparisons than for most other EV source dimensions. Fourteen studies explicitly included both BM- and AD-MSC EVs, including four comparisons that also incorporated an umbilical MSC source, although these studies addressed different molecular, cellular, and disease endpoints. This larger evidence base consistently supports the general conclusion that MSC tissue source can influence EV composition or biological activity, but it does not establish a uniform hierarchy of therapeutic superiority. Relative performance varied with recipient cell, disease model, and endpoint; notably, a 2026 UC- versus AD-MSC comparison reported biologically distinct but clinically convergent skin-regeneration outcomes [12]. Not all regenerative comparisons were concordant. In a 2021 cartilage- and bone-regeneration study, adipose-derived MSC EVs showed greater regenerative activity than bone marrow- and synovial-MSC EVs, whereas a 2026 craniofacial bone-regeneration study reported superior osteogenic performance of bone marrow-derived MSC EVs compared with adipose-derived EVs [48,49]. These opposing source rankings arose in related but non-identical experimental settings and were therefore interpreted as discordant/context-dependent evidence rather than a proof of universal superiority of either source. Thus, evidence is comparatively consistent for source-dependent heterogeneity but mixed or context-dependent regarding which MSC tissue origin is therapeutically superior (Figure 5).

#### 3.9.4. Neural Stem/Progenitor and Mature Neural-Cell EVs

The therapeutic goals of neural EV therapy differ fundamentally from cytotoxic immune EV therapy. Relevant outcomes include neuronal survival, axonal and synaptic support, neuroinflammation, microglial phenotype, blood–brain barrier integrity, white-matter preservation, neurovascular repair, and functional recovery (Figure 6).

Human neural-stem-cell EVs improved tissue and functional outcomes in a porcine model of ischemic stroke, providing an important large-animal demonstration of neuroprotective potential [51]. Neural-progenitor-cell EVs have also been shown to preserve photoreceptors in retinal degeneration through microglial inactivation associated with an anti-inflammatory miRNA network [40].

Direct neurotherapeutic source comparisons remain distributed across heterogeneous source pairs, recipient systems, and endpoints, with few repeated matched comparisons of the same candidate sources. The available studies therefore support source- and state-dependent neural EV behavior but do not yet establish a consistent cross-study ranking among NSC/NPC-, iPSC-neural-, and MSC-derived platforms. Further matched evaluation of these sources would help define their relative neurological therapeutic profiles.

Mature neural-cell EVs require even greater caution. Astrocyte-derived EVs can be protective or pro-inflammatory depending on the stress state of the parent cell; a 2025 study demonstrated a double-edged effect of hypoxia/reoxygenation duration on the astrocyte EV phenotype [50]. Pathological neurons or glia may also transmit disease-associated signals. Thus, the state of the parent cell may be as important as its lineage.

#### 3.9.5. Cardiac, Vascular, and Pluripotent-Cell EVs

Cardiac-lineage EVs include vesicles from cardiac progenitors, cardiomyocytes, epicardial cells, fibroblasts, and vascular/endothelial progenitors. Their biological rationale includes myocardial survival, angiogenesis, antifibrotic signaling, and tissue-specific regenerative communication.

A 2024 head-to-head comparison evaluated sEVs from primary BM-MSCs, immortalized MSCs, ESCs, ESC-derived cardiac progenitors, ESC-derived cardiomyocytes, and ventricular cardiac fibroblasts. ESC-derived sEVs showed the most favorable overall proangiogenic and antifibrotic performance in vitro and produced the most favorable outcome among selected candidates in a myocardial ischemia–reperfusion model [6]. This apparent ESC-sEV advantage derives from a single six-source head-to-head study. Other cardiovascular comparisons in the direct-comparison inventory involved different source pairs and experimental contexts; accordingly, this ranking should not be interpreted as an independently replicated or universal hierarchy for cardiac repair.

Epicardial EVs provide another tissue-specific example: conserved miRNA transfer has been associated with cardiomyocyte proliferation and regenerative signaling [52]. Endothelial progenitor-cell EVs can support angiogenesis and tissue repair, especially when delivery is sustained [53]. The same angiogenic specialization, however, may be inappropriate where pathological neovascularization is undesirable.

### 3.10. Direct Head-to-Head Evidence: The Core Evidence Layer

The most informative evidence for rational source selection comes from direct head-to-head studies in which EVs from two or more sources are prepared using comparable isolation approaches and evaluated under substantially matched experimental conditions using common outcome assessments. Such designs reduce, although they do not eliminate, methodological confounding between the EV source and observed biological effects, but they remain uncommon relative to single-source proof-of-concept studies (Table 4).

Across the complete 70-study inventory, the number of studies contributing to individual source-level conclusions was highly uneven. Recurrent direct comparisons were concentrated among MSC source families, whereas several immune-effector, neural, engineered, and cardiac source rankings were supported by only one or a small number of matched studies. Across repeated comparisons, the most consistent higher-level finding was that parental-cell source can influence EV composition or biological performance; in contrast, the identity of the best-performing source frequently varied with the target cell, disease model, endpoint, and experimental conditions. Apparent differences in source ranking across non-equivalent models were therefore interpreted as context-dependent findings rather than automatically as contradictions, whereas materially opposing results from sufficiently comparable studies were considered discordant evidence. For example, regenerative comparisons yielded opposing BM-MSC versus AD-MSC source rankings across bone-related models: one study favored adipose-derived EVs for cartilage and bone regeneration, whereas a later craniofacial bone-regeneration study favored bone marrow-derived EVs [48,49]. Such discrepancies were retained in the synthesis rather than resolved by selectively emphasizing the favorable study.

### 3.11. Advantages, Disadvantages, and Context-Dependent Trade-Offs

A central principle of this review is that the same biological property can be beneficial in one indication and harmful in another. The purpose of disadvantage analysis is therefore not simply to exclude a source, but to identify predictable liabilities that can be avoided through better source selection, cell-state control, vesicle-fraction selection, engineering, dosing, or route design (Table 5).

### 3.12. Comparative Evidence Dimensions

The comparative synthesis separates three distinct dimensions of the evidence: comparative confidence (CEC), Translational Development Stage (TDS), and strength of cargo–mechanism–function evidence (CMEC). Source-level assignments were made according to the operational criteria defined in Section 2.6. These classifications are qualitative evidence-mapping descriptors and should not be interpreted as validated quantitative, risk-of-bias, or certainty-of-evidence scores. These source-level descriptors represent a secondary interpretive mapping applied after assembly of the primary comparative evidence layer and are not components of the original study-selection process. CEC should therefore be interpreted together with the descriptive depth and consistency of the underlying direct-comparison literature: the same CEC assignment does not imply an identical number of supporting studies, and repeated context-dependent findings were not considered equivalent to repeated concordant evidence for a specific superiority claim.

### 3.13. Functional Identity and Best-Fit Applications

Functional profiles differed substantially across EV sources (Table 6). Immune-cell-derived EVs were primarily associated with cytotoxic or immunoregulatory functions, whereas MSC-, neural-, cardiac-, and pluripotent-cell-derived EVs were predominantly linked to regenerative, anti-inflammatory, neuroprotective, or tissue-repair applications.

### 3.14. Disadvantages, Evidence Strength, and Translational Development Stage

Application of the operational qualitative evidence-mapping criteria demonstrated substantial differences among EV sources in comparative evidence strength, translational development stage, and mechanistic support (Figure 7). Importantly, these dimensions did not necessarily progress together; an EV source could have relatively strong mechanistic support while remaining clinically immature, or it could have entered clinical evaluation without evidence of superiority over alternative sources. The TDS descriptor captures the highest documented development stage rather than overall therapeutic readiness. Human administration therefore increases the documented translational stage but does not imply that manufacturing reproducibility, potency, safety, biodistribution, batch consistency, or efficacy requirements have been satisfied.

### 3.15. Qualitative Evidence Mapping and Cargo–Mechanism Support

Cargo identification or enrichment alone was therefore treated as associative evidence and was not considered sufficient to establish a causal cargo–mechanism–function relationship. Comparative confidence, translational development stage, and mechanistic confidence were evaluated as separate qualitative dimensions using the operational criteria defined in Section 2.6. This distinction avoids equating strong biological activity with strong comparative evidence or advanced clinical translation. A source may therefore be mechanistically compelling but clinically immature or be clinically evaluated without demonstrated superiority over competing sources (Figure 8).

### 3.16. Comparative Evidence Category (CEC)

Application of the criteria defined in Section 2.6 categorized comparative evidence from CEC-A (strongest comparative support) to CEC-D (emerging evidence). Table 7 summarizes the operational definitions used for these assignments.

### 3.17. Translational Development Stage, Clinical Efficacy, and Comparative Clinical Evidence

For evidence mapping, TDS was used to record the highest clearly documented stage of translational development, ranging from mechanistic or in vitro evidence (TDS-0) to Phase II or later therapeutic evaluation (TDS-4). TDS therefore records developmental progression only and should not be interpreted as therapeutic readiness. Clinical efficacy was interpreted separately and required evidence of therapeutic benefit in human studies, whereas comparative clinical evidence required direct clinical comparison between EV products or source categories. Consequently, a high TDS assignment based on Phase I/II evaluation does not imply demonstrated efficacy or comparative clinical superiority.

### 3.18. Cargo–Mechanism Evidence Category (CMEC)

CMEC assessed mechanistic confidence independently of comparative evidence and TDS, using the operational criteria defined in Section 2.6. CMEC-0 indicated no demonstrated cargo–mechanism linkage; CMEC-1 indicated candidate cargo or pathway association without experimental perturbation; CMEC-2 indicated perturbational evidence supporting the relationship between cargo/pathway and EV-mediated function; and CMEC-3 required strong causal linkage supported by direct manipulation and additional confirmatory evidence (Figure 8).

### 3.19. EV-FIT+: Function, Indication, Trade-Off, and Contra-Function

EV-FIT+ is an author-developed qualitative conceptual framework that emerged during the interpretive synthesis of this review after recurrent relationships among EV source, therapeutic function, indication-specific suitability, trade-offs, and contra-functions became apparent. It was not prospectively predefined and was not used for literature searching, study eligibility, or construction of the primary direct-comparison evidence layer. EV-FIT+ is intended to structure source-to-indication fit and should not be interpreted as a validated quantitative scoring system, predictive model, or clinical decision instrument. The EV-FIT+ framework begins with the clinical requirement rather than the cell source available in a laboratory (Table 8). Its purpose is to structure EV source selection around therapeutic fit (Figure 9). Within EV-FIT+, this therapeutic fit corresponds to the operational definition of therapeutic suitability used in this review: a concordance between the required therapeutic function and the source/product’s demonstrated function, considered together with potency and mechanistic support, indication-specific risks and contra-functions, manufacturing feasibility, and translational evidence. EV-FIT+ is intended to complement, rather than replace, existing EV characterization, potency, and translational frameworks. Whereas these approaches primarily address how an EV product should be characterized, functionally assessed, manufactured, and advanced toward translation, EV-FIT+ adds an indication-first source-selection layer by explicitly matching the required therapeutic function to source-specific trade-offs and contra-functions before considering potency, safety, manufacturing, and translational development.

### 3.20. Evidence Gaps and Why Definitive Rankings Are Premature

Current evidence is insufficient for definitive source ranking across most indications because both the volume and comparability of direct evidence are uneven. Although 70 direct source-comparison studies were identified overall, 52 included at least one MSC source, whereas several immune-effector, neural, cardiac, and engineered source comparisons were represented by only one or a few matched studies. Evidence gaps therefore reflect both unequal study density across source families and a lack of standardized comparative design (Figure 7).

First, most studies evaluate one EV source without a matched comparator. Second, isolation methods differ substantially, including ultracentrifugation, precipitation, size-exclusion chromatography, filtration, density gradients, and combined methods. Third, dose normalization is inconsistent, relying variously on total protein, particle number, conditioned-medium volume, or parent-cell equivalents. Fourth, parent-cell state is frequently underreported, despite evidence that hypoxia, cytokines, senescence, differentiation, and stress can materially alter EV phenotype. Fifth, conventional EV identity does not substitute for a mechanism-linked potency assay [1,5].

Consequently, a literature-based ranking can reflect both methodological differences and uneven evidence density rather than true biological superiority. Where related studies produced different source rankings under different recipient cells, models, doses, or endpoints, these findings were interpreted as context-dependent rather than automatically contradictory; only materially opposing findings from sufficiently comparable experimental settings were considered discordant.

### 3.21. Risk-Adjusted Source Selection: Using Disadvantages to Improve EV Design

Source-specific disadvantages should be used proactively. A procoagulant EV fraction does not necessarily require abandoning the parent-cell source; it may instead justify fraction selection, TF/CD142 testing, a coagulation assay, lower dose, slower infusion, or a non-intravenous route [46].

Similarly, insufficient NK-EV tumor specificity may motivate CAR engineering or post-isolation targeting. CAR-EV antigen escape may motivate multi-antigen targeting or combination with broader NK-derived cytotoxic EVs. Weak DC-EV direct cytotoxicity is not a defect when the therapeutic goal is vaccination, but it may motivate combination with an effector EV when both immune instruction and immediate killing are required.

For neural disease, the manufacturing complexity of NSC/NPC EVs should trigger direct comparison against more scalable UC/WJ- or iPSC-derived neural-support platforms before a manufacturing program is committed. For MSC-derived EVs, broad regenerative activity should be accompanied by indication-specific screens for unwanted angiogenesis, immunosuppression, fibrosis, coagulation, or tumor-supportive signaling.

### 3.22. Minimum Dataset for Future Head-to-Head EV Studies

A minimum reporting framework was proposed for future head-to-head EV studies, covering parent-cell characteristics, EV preparation, functional assessment, and translational variables to improve comparability, reproducibility, and clinical relevance (Table 9).

These recommendations align conceptually with MISEV2023 and with calls for functional, mechanism-relevant assays of therapeutic EV products [1,5].

## 4. Discussion

The available evidence supports treating therapeutic EVs as source- and product-specific biological preparations rather than as a homogeneous therapeutic class. Functional differences can arise independently from parental-cell lineage, anatomical tissue origin, parental-cell engineering status, cellular state, vesicle biogenesis and cargo sorting, manufacturing, and the recipient biological context. Accordingly, differences attributed to “source” were interpreted, where study design permitted, as cell-lineage effects, tissue-of-origin effects, or engineered parental-cell effects rather than as a single undifferentiated source variable. This is illustrated by the contrasting immune functions of cytotoxic T-cell and Treg EVs, source-dependent differences among MSC EVs, and state-dependent changes in astrocyte EV phenotype [3,4,37,38,50]. The comparative literature also contains discordant findings that argue against simple source rankings. A notable example is the BM-MSC versus AD-MSC comparison in skeletal regeneration: adipose-derived EVs showed superior activity in one cartilage/bone-regeneration model, whereas a later craniofacial bone-regeneration study favored bone marrow-derived EVs [48,49]. Together with studies showing clinically convergent outcomes despite biological differences between sources [12], these findings reinforce that apparent source superiority may depend on the target tissue, recipient cell, disease model, and experimental conditions rather than representing an invariant property of the parental-cell source.

These differences have direct implications for therapeutic selection. Cytotoxicity, immunosuppression, angiogenesis, and antigen specificity can each represent either a desirable function or a liability depending on the indication. EV-FIT+ therefore organizes source selection around required function, indication-specific trade-offs, and contra-functions. Safety should likewise be interpreted at the product level: a signal observed with one preparation should not be generalized to all EVs from the same parental-cell source. The tissue factor-associated coagulation signal observed in selected UC-MSC EV fractions illustrates one such product-specific hazard, but a broader risk framework should also consider immunogenicity and immunotoxicity, biodistribution and clearance, non-target organ accumulation, residual process- or source-derived contaminants, co-isolated non-vesicular material and protein aggregates, and repeat-dose or longer-term toxicity. These dimensions should be evaluated in relation to vesicle fraction, isolation/manufacturing method, dose, route, and duration of exposure [1,54,55,56].

Biodistribution is particularly relevant to product safety because systemically administered EVs frequently distribute to the liver, lungs, spleen, and kidneys, although the pattern varies with vesicle size, source, dose, administration route, and detection method. Such non-target organ exposure may influence both pharmacological activity and toxicity and should therefore be assessed alongside clearance and persistence. Likewise, residual manufacturing reagents, media-derived components, lipoproteins or other non-vesicular particles, protein aggregates, and storage-related degradants may alter biological activity or introduce risks that cannot be attributed to the EV source itself [1,54,56].

A major methodological barrier remains heterogeneity across studies in EV isolation, characterization, dose normalization, recipient models, and outcome assessment. Harmonized comparative studies using transparent EV characterization, orthogonal exposure metrics, and mechanism-linked potency assays would improve the attribution of observed effects to the EV source [1,5]. Recent historical analysis of the field similarly emphasizes methodological standardization, consensus nomenclature, and improved isolation and characterization as important for reproducibility and translation [57].

A second challenge is distinguishing translational development stage, clinical maturity, therapeutic efficacy, and comparative clinical evidence. Large-animal evaluation, GMP-compatible production, and progression to human Phase I/II studies are important developmental milestones, but they do not establish therapeutic efficacy. Clinical maturity reflects accumulated experience with manufacturing, administration, feasibility, and safety in humans, whereas therapeutic efficacy requires evidence of clinically meaningful patient benefits. Comparative clinical evidence requires an additional level of evidence in which competing EV products or source categories are directly compared in a clinical setting. DC-derived EV preparations have progressed to Phase I/II evaluation and therefore have greater clinical development experience than many NK, CAR-T, CAR-NK, neural, and engineered platforms [43,44], but this does not establish comparative clinical superiority. Similarly, early human administration of UC-MSC-derived EV preparations provides preliminary clinical and safety experience, while controlled efficacy and comparative clinical evidence remain limited [45,46].

This narrative review has several methodological limitations. Although we conducted an extensive reproducible electronic search of PubMed/MEDLINE, Europe PMC, and OpenAlex, the search did not include Embase, Scopus, or Web of Science Core Collection because complete reproducible exports were not available. Consequently, some relevant studies may have been missed, particularly given the multidisciplinary nature of EV research. Google Scholar was used as a supplementary source for targeted searching and citation tracking because its approximate result counts and ranking are insufficiently stable for use as a primary source for numerical search reporting. Screening and structured data extraction for the primary direct-comparison evidence layer were conducted by a single reviewer and were not independently duplicated. This introduces a potential risk of study-selection and data-extraction errors, as well as reviewer-dependent judgment when interpreting heterogeneous source comparisons. Although the subsequent source-level CEC, TDS, and CMEC assignments were independently rechecked by two authors, this secondary verification does not substitute for independent duplicate screening and data extraction. In addition, the direct-comparison literature is methodologically heterogeneous in EV isolation, characterization, dose normalization, source definition, recipient models, and outcome measures, precluding quantitative meta-analysis and a single cross-design risk-of-bias instrument.

The resulting synthesis should therefore be interpreted as a transparent comparative evidence map rather than a pooled estimate of relative efficacy. Similarly, the descriptive numbers of studies contributing to individual narrative conclusions indicate evidence depth rather than statistical weight. These counts do not account for differences in experimental scale, precision, effect magnitude, methodological quality, or comparability, and a larger number of heterogeneous studies should not be interpreted as inherently stronger evidence than a smaller number of well-matched comparisons.

CEC, TDS, CMEC, and EV-FIT+ were developed during interpretive synthesis rather than prospectively specified before the literature search and should therefore be distinguished from the primary direct-comparison evidence synthesis. CEC, TDS, and CMEC should also be interpreted as author-developed qualitative evidence-mapping descriptors rather than validated grading instruments. Although explicit operational criteria were applied to improve transparency and consistency, the categories inevitably involve some interpretive judgment and should not be equated with formal risk-of-bias, certainty-of-evidence, or regulatory-readiness assessments.

### 4.1. Extracellular Vesicles as Drug-Delivery Platforms: Carrier Selection Is Also Source-Dependent

Beyond their use as intrinsically bioactive cell-free therapeutics, EVs can also be exploited as natural drug-delivery vehicles for small molecules, proteins, nucleic acids, gene-editing components, and other therapeutic payloads. Their lipid bilayer can protect cargo from extracellular degradation, while their biological membranes provide cellular uptake mechanisms and, in some settings, source- or surface-dependent tropism. This distinction is important for clinical interpretation: an EV product may act through its endogenous cargo, through an intentionally loaded therapeutic payload, or through both. Treating these modes separately can make the therapeutic mechanism and potency assay more explicit and may reduce some of the uncertainty associated with broadly defined ‘exosome therapy,’ although it does not remove the need for rigorous characterization, safety testing, and manufacturing control [58,59,60,61].

Among the sources discussed in this review, MSC-derived EVs currently appear to be among the more pragmatic general-purpose delivery chassis. Their appeal reflects scalable parental-cell expansion, relatively high EV production, and extensive experience with loading or delivering chemotherapeutic agents, siRNA, miRNA, and other cargo. Although MSC-derived EVs are often considered to have a favorable immunogenicity profile, unintended immune recognition and clearance remain incompletely characterized and should not be assumed to be negligible [55]. For allogeneic manufacturing, UC/WJ-MSC-derived EVs may be particularly attractive because of source accessibility and expansion potential; however, direct evidence that UC/WJ EVs are intrinsically better drug carriers than BM- or adipose-MSC EVs remains insufficient. In oncology, immune-derived vesicles such as NK-, CAR-T/CAR-NK-, or DC-derived EVs may offer more specialized targeting or immunological functionality, but their manufacturing maturity and cross-source comparative evidence are less developed. Thus, the optimal carrier source should be selected according to payload, target tissue, route, desired native function, and acceptable trade-offs rather than by source reputation alone [59,62,63,64].

The EV-FIT+ framework can therefore be extended to drug delivery. In this setting, Function includes both the activity of the loaded drug and the native biological activity of the carrier; Contra-Function includes undesired endogenous EV signaling, off-target uptake, procoagulant activity, or immune effects. Source selection should consequently consider loading efficiency, cargo stability, release kinetics, biodistribution, target-cell uptake, residual endogenous cargo, scalability, batch reproducibility, and route-specific safety. This carrier-based perspective broadens the translational role of EVs: a source whose endogenous therapeutic effect is modest may still become valuable if it provides a safe, scalable, and target-compatible delivery vehicle. Conversely, a biologically potent EV source may be a poor carrier if its intrinsic cargo introduces an unwanted contra-function [58,60,61,64,65].

### 4.2. Research Priorities

#### 4.2.1. Immune Effector EV Challenge

Compare primary NK, activated/memory-like NK, CD8/CTL, γδ-T, CAR-T, CAR-NK, and DC EVs using identical tumor models, EV isolation, dose normalization, and endpoints including direct killing, antigen specificity, T-cell priming, resistance to suppressive microenvironments, biodistribution, and off-target toxicity.

#### 4.2.2. Neuroregenerative EV Challenge

Compare NSC/NPC, iPSC-neural, UC/WJ-MSC, placental-MSC, BM-MSC, and AD-MSC EVs using neuronal survival, neurite extension, synaptic recovery, microglial phenotype, BBB integrity, lesion volume, white-matter preservation, and functional recovery.

#### 4.2.3. Regenerative/Inflammatory EV Challenge

Compare UC/WJ, placental, BM, AD, Treg, and regulatory macrophage EVs using inflammatory suppression, macrophage polarization, angiogenesis, epithelial repair, fibroblast activity, fibrosis, and contra-function screens.

#### 4.2.4. Cross-Source Safety Challenge

Apply a common product-level safety panel to systemically administered EV products that includes coagulation/TF activity; immunogenicity, complement activation, and cytokine release; biodistribution, clearance, and non-target organ accumulation; residual process- and source-derived contaminants, non-vesicular co-isolates, and protein aggregates; unwanted cytotoxic, proangiogenic, tumor-supportive, or immunosuppressive activity; and repeat-dose and longer-term toxicity [1,54,55,56].

## 5. Conclusions

Within the limitations of the available literature, comparative evidence suggests that EV cargo, phenotype, biological activity, therapeutic performance, and risk vary according to cell source, tissue origin, engineering status, and cellular state. However, uneven evidence density and substantial heterogeneity in EV isolation, characterization, dosing, experimental models, and outcome assessment preclude definitive cross-source rankings. The present synthesis should therefore be interpreted as a comparative evidence map rather than as evidence of a universal hierarchy of EV superiority.

We propose a qualitative source-to-fit framework for therapeutic EV development: [Cell Source + Tissue/Anatomic Origin + Parental-Cell Engineering Status] → State → Vesicle Identity → Cargo → Function → Indication → Trade-off/Contra-Function → Potency → Safety → Translational Readiness. This framework is intended to support indication-specific interpretation and hypothesis generation rather than serve as a validated predictive or clinical decision tool. Translational readiness should be assessed at the product level, and clinical development stage, therapeutic efficacy, and comparative clinical evidence should be considered separately. More standardized and replicated head-to-head studies are needed before firm conclusions regarding therapeutic superiority can be drawn.

## Figures and Tables

**Figure 1:**
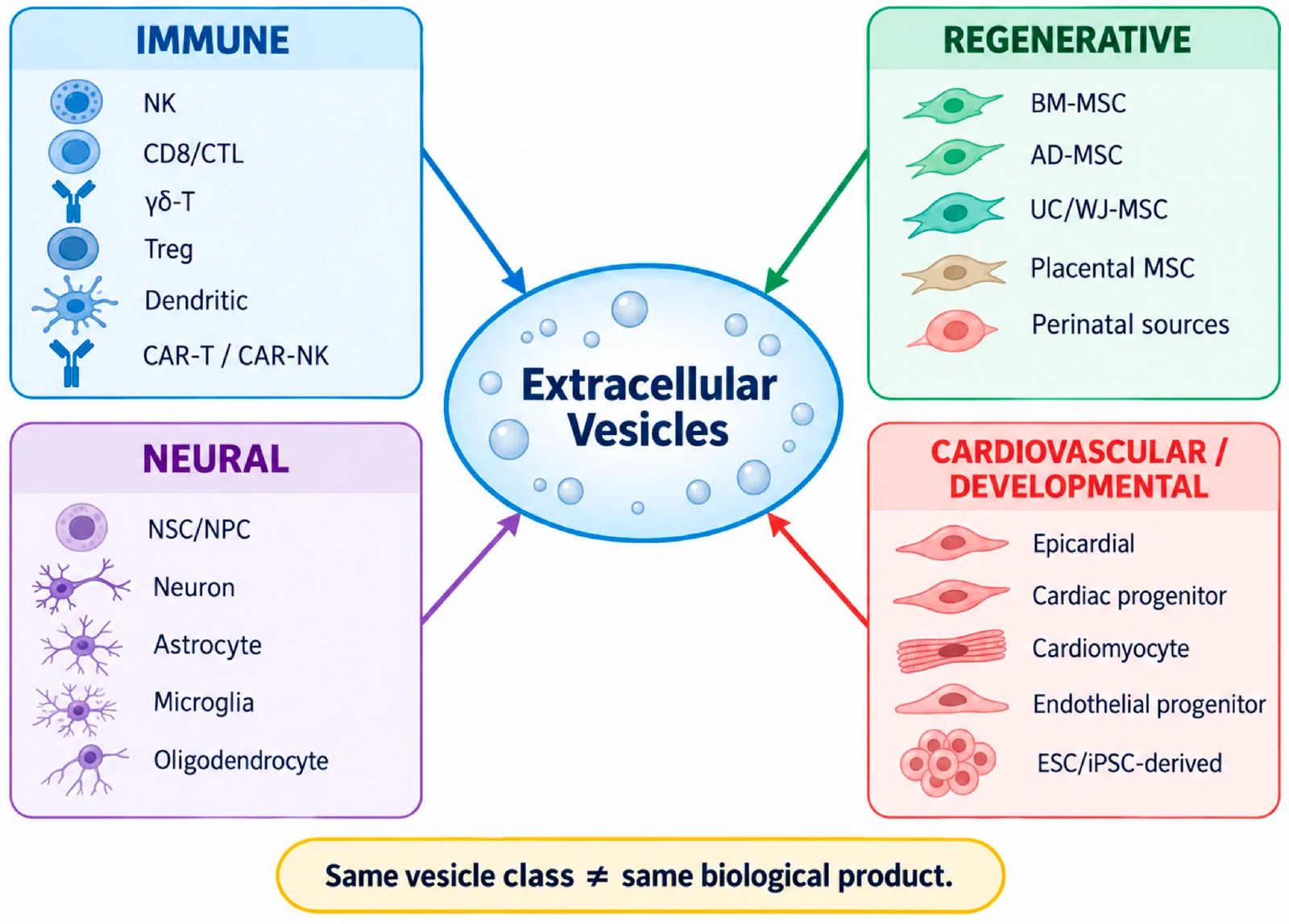
The EV source universe. Therapeutically relevant EV sources can be organized into immune-effector, regenerative/stromal, neural, and cardiovascular/developmental families. These families nevertheless contain biologically heterogeneous EV preparations.

**Figure 2:**
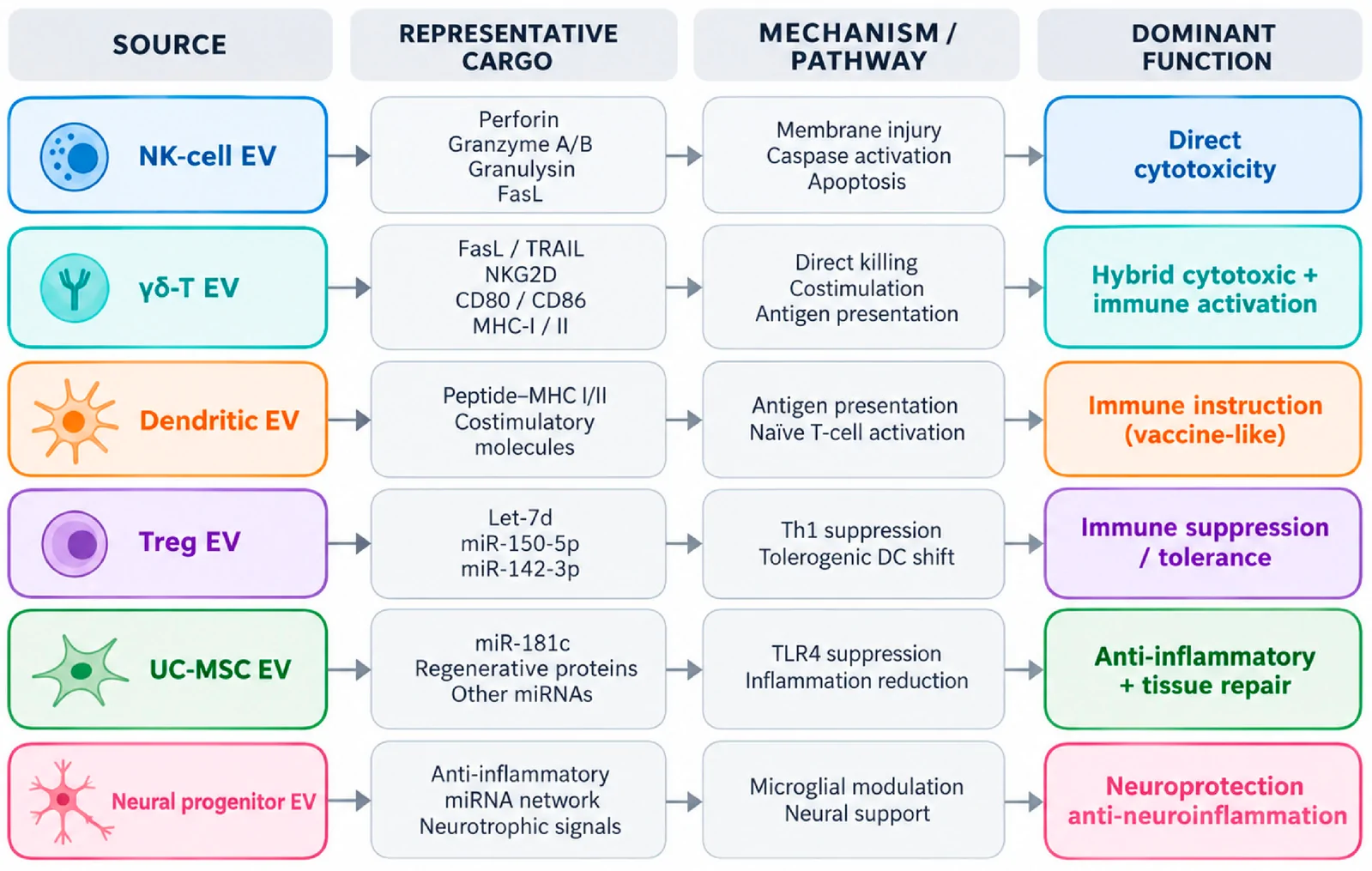
Source-to-cargo-to-function atlas. Representative experimentally reported cargo illustrates source-associated molecular differences and candidate cargo–function relationships. The presence or enrichment of a cargo molecule should not be interpreted as evidence that it causally mediates the observed biological effect unless supported by appropriate mechanistic experiments. The cargo shown is illustrative rather than an invariant signature of every preparation from that source; the strength of the cargo–mechanism evidence is considered separately using CMEC [2,34,35,36,37,38,39,40].

**Figure 3:**
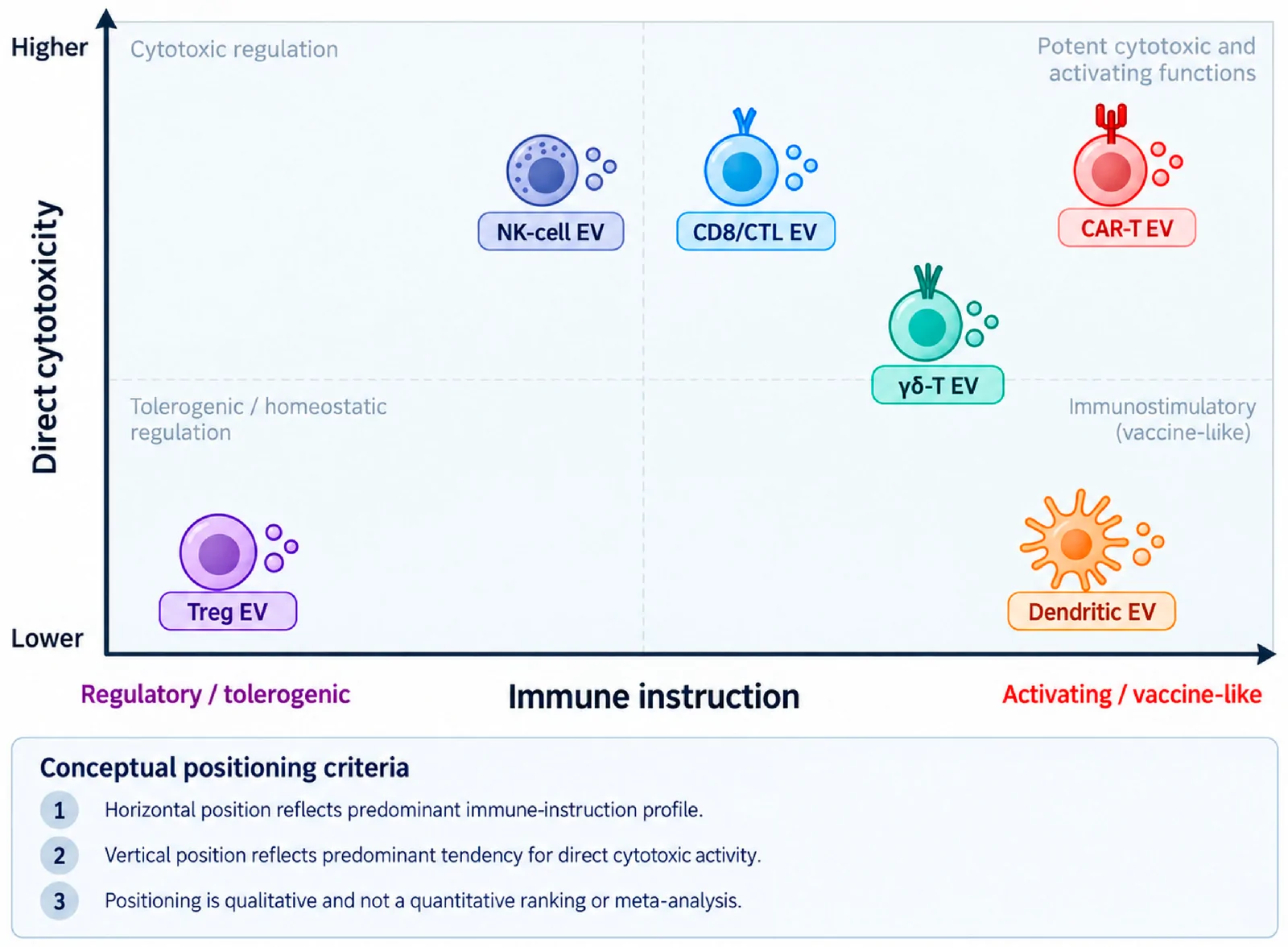
Immune-EV functional spectrum. Horizontal positioning reflects the predominant reported immune-instruction phenotype, ranging from regulatory/tolerogenic to activating or vaccine-like activity, whereas vertical positioning reflects the predominant tendency for direct cytotoxic activity. Positions are qualitative summaries intended for mechanistic comparison; distances between sources do not represent quantitative effect sizes, pooled estimates, or therapeutic rankings.

**Figure 4:**
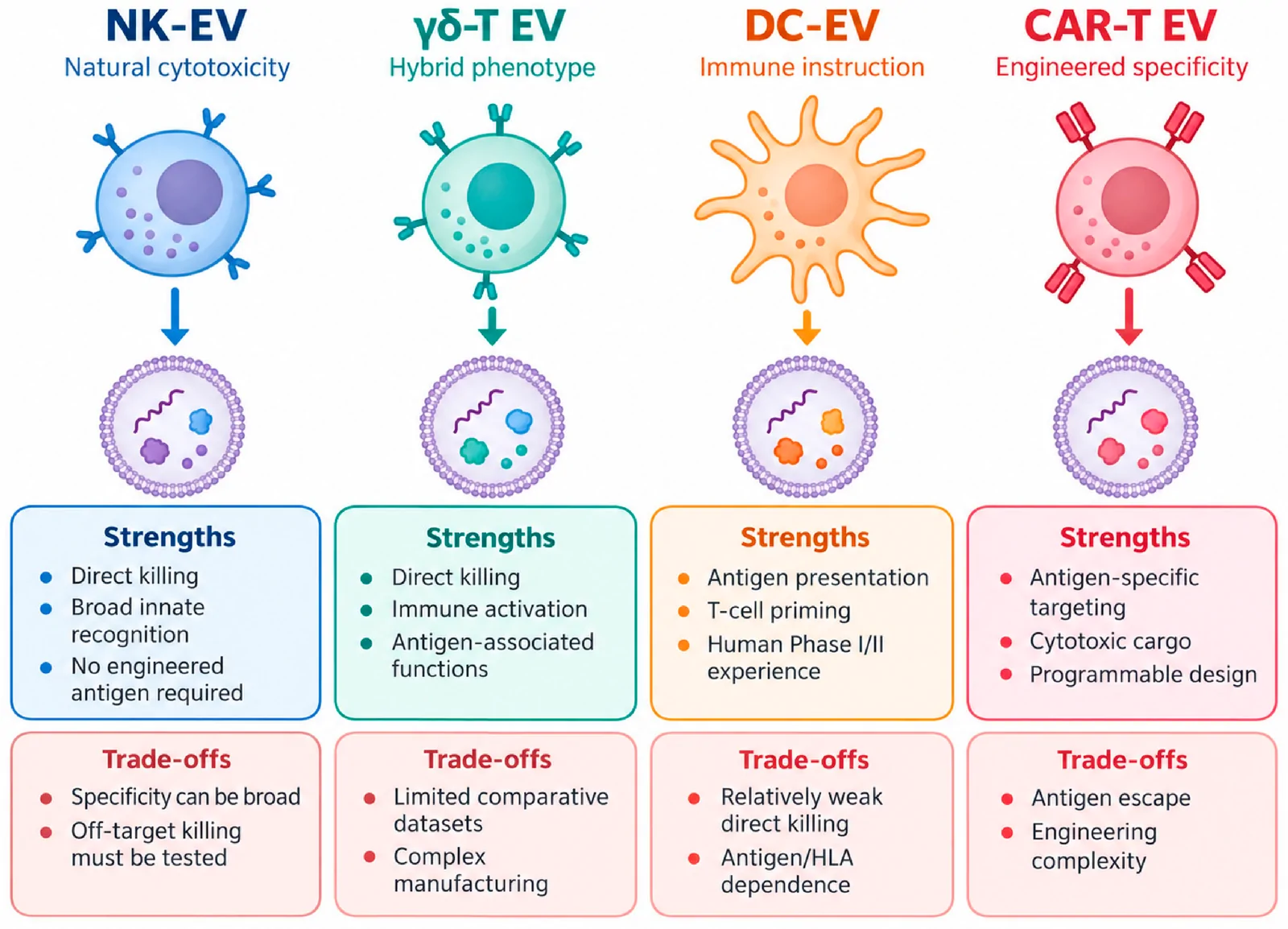
Distinct EV strategies for tumor control. NK-EVs emphasize natural cytotoxicity, γδ-T EVs can combine killing and immune activation, DC-EVs emphasize antigen presentation, and CAR-T EVs emphasize engineered antigen specificity. Each strategy carries distinct disadvantages and manufacturing trade-offs [2,34,35,36,43,44].

**Figure 5:**
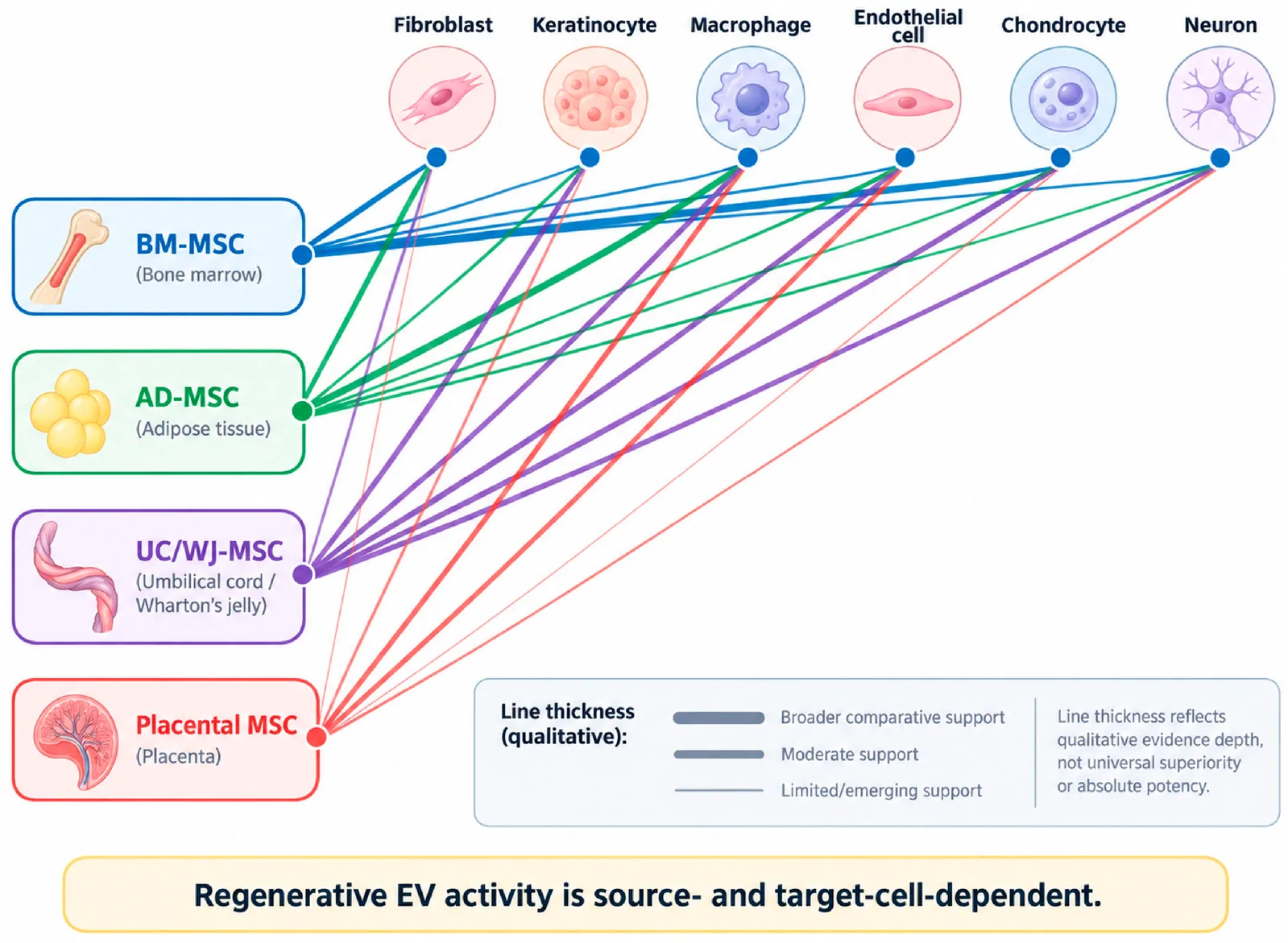
Regenerative EV source map. BM-MSC-, AD-MSC-, UC/WJ-MSC-, and placental-MSC-derived EVs have been evaluated across different repair-relevant target cells. Connections indicate reported source–target cell relationships identified in the reviewed literature and do not represent effect magnitude, potency, evidence strength, or universal therapeutic superiority [3,4,7,8,39,47].

**Figure 6:**
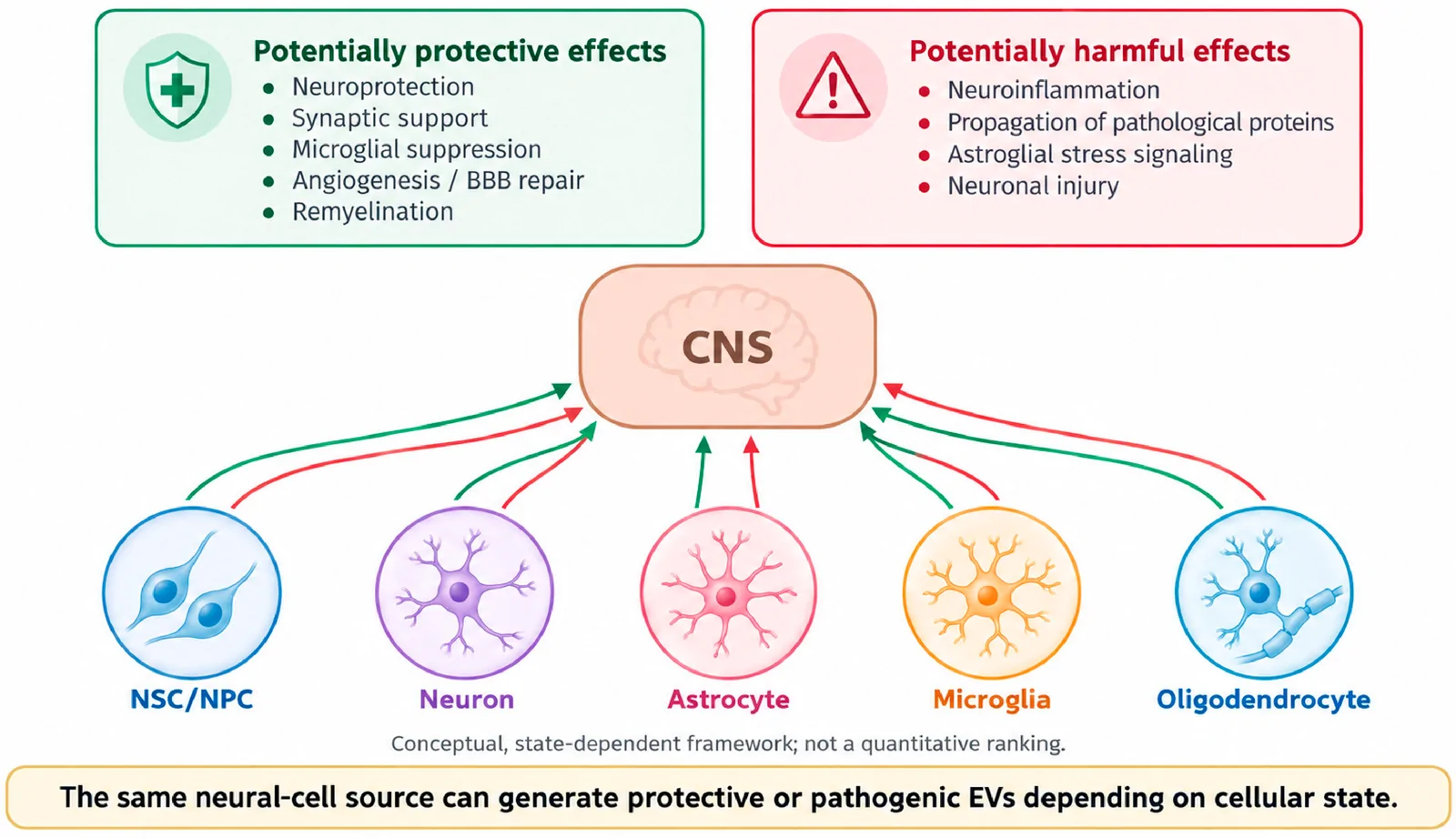
Neural EV landscape. Neural stem/progenitor, neuronal, astrocytic, microglial, and oligodendroglial EVs may support neuroprotection or propagate pathological signals depending on cellular state and disease context. The figure emphasizes that source labels alone are insufficient for neural EV product definition [40,50,51].

**Figure 7:**
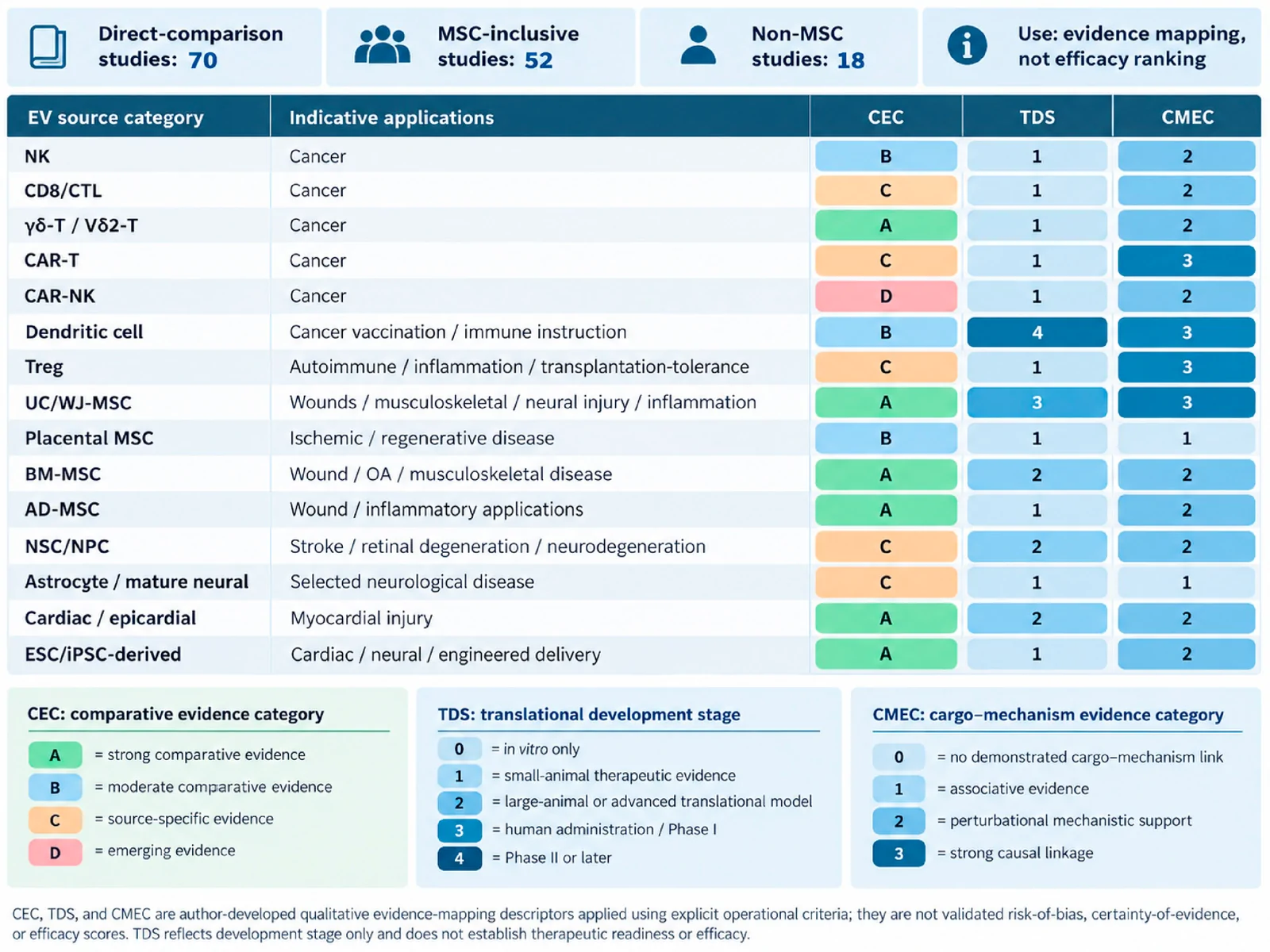
Quantitative evidence landscape of therapeutic EV sources. The figure summarizes source-level comparative evidence using the 70-study direct-comparison layer together with the author-developed Comparative Evidence Category (CEC), Translational Development Stage (TDS), and Cargo–Mechanism Evidence Category (CMEC). CEC reflects the strength of comparative support, TDS indicates the highest documented stage of translational development, and CMEC reflects mechanistic confidence. These descriptors are intended for evidence mapping and should not be interpreted as pooled effect estimates, therapeutic efficacy rankings, or validated certainty-of-evidence scores.

**Figure 8:**
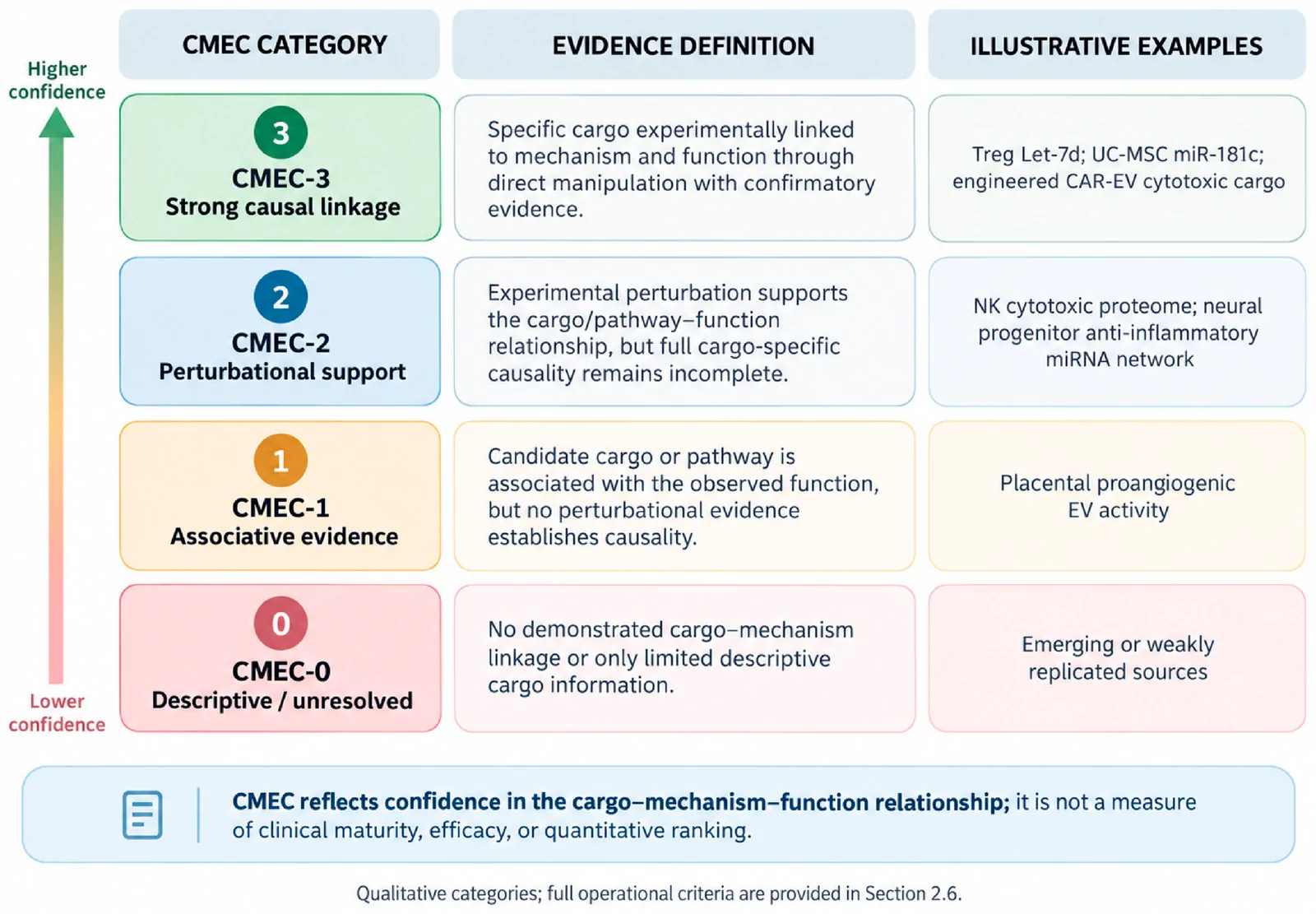
CMEC represents increasing confidence in the cargo–mechanism–function relationship: CMEC-0, no demonstrated cargo–mechanism linkage; CMEC-1, candidate cargo/pathway association without perturbational evidence; CMEC-2, experimental perturbation supporting the cargo/pathway–function relationship; CMEC-3, strong causal linkage supported by direct manipulation and additional confirmatory evidence. Full operational criteria are provided in Section 2.6.

**Figure 9:**
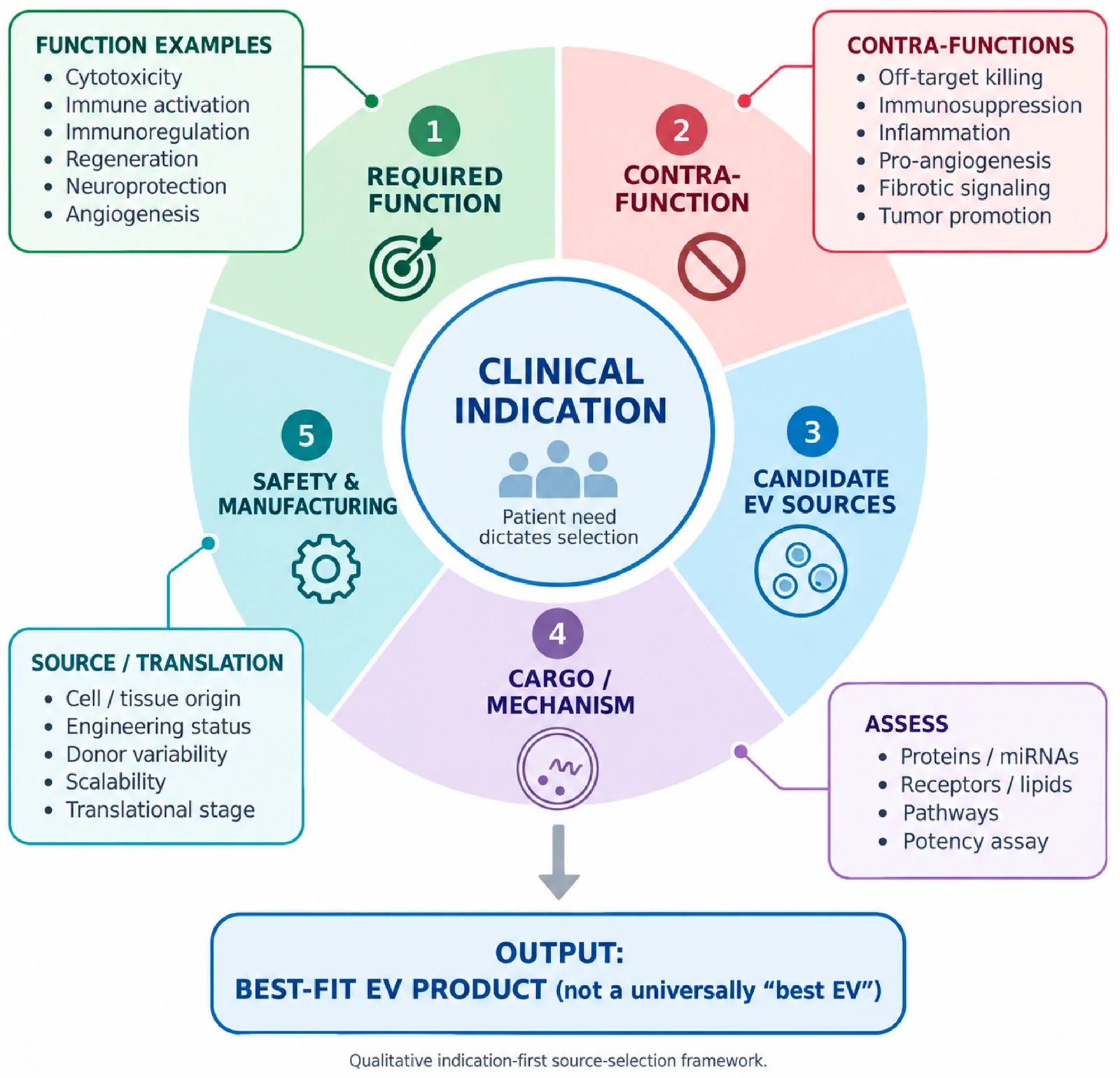
EV-FIT+ qualitative source-selection framework. The process begins with the required therapeutic function and explicitly identifies contra-functions that would be harmful in the target indication. Candidate sources are then evaluated for cargo/mechanism, potency, safety, manufacturing, and translational development stage before selecting a best-fit EV product.

**Table 1:** Eligibility, selection, and extraction framework for the comparative EV evidence synthesis.

Domain	Included	Excluded/Down-Weighted
EV identity	Source-defined mammalian EVs/sEVs, including preparations termed ‘exosomes’ by the original studies with identifiable parental cell or tissue source.	Non-EV secretome only; source not identifiable; vesicle identity inseparable from other products.
Evidence contribution	Cargo/phenotype, mechanism, therapeutic function, source comparison, safety, manufacturing, or translational evidence.	Purely diagnostic biomarker studies without therapeutic source-to-function relevance.
Comparative priority	Direct head-to-head source comparisons using the same or methodologically comparable EV isolation/purification approach, substantially matched experimental conditions, and common outcome assessment received the greatest interpretive weight.	Comparisons in which source effects could not be distinguished from major differences in EV isolation, experimental conditions, model systems, or outcome assessment were excluded from the direct-comparison layer or interpreted only as contextual evidence.
Study design	In vitro, in vivo, large-animal, human, and relevant engineering/manufacturing studies.	Editorials or commentaries without primary evidence, except methodological guidance used for reporting context.
Data extracted	Cell type/lineage, tissue/anatomic origin, parental-cell engineering status, donor/cell state, vesicle fraction, comparator design, cargo, target, mechanism, function, indication, trade-offs, safety, manufacturing, in vivo/translational evidence, and highest documented translational stage.	Claims that could not be linked to a defined EV source or interpretable experimental context.

Abbreviations: EV, extracellular vesicle; sEV, small extracellular vesicle.

**Table 2:** Evidence hierarchy used for comparative interpretation.

Tier	Evidence Type	Interpretation
Tier I	Direct head-to-head evidence	Two or more EV sources compared within the same study using the same or methodologically comparable isolation/purification approach, substantially matched experimental conditions, and at least one common outcome assessed by the same method.
Tier II	Disease-matched comparative evidence	Different sources evaluated in similar disease contexts but not within the same experiment.
Tier III	Mechanistic evidence	Cargo, receptors, signaling pathways, recipient-cell effects, and causality studies.
Tier IV	Translational evidence	Large-animal studies, human administration, clinical trials, GMP-compatible or scalable manufacturing.

Abbreviations: EV, extracellular vesicle; GMP, Good Manufacturing Practice.

**Table 3:** Functional characteristics and representative sources of therapeutic EVs.

Functional Family	Representative Sources	Dominant Therapeutic Phenotype
Cytotoxic immune-effector EVs	NK, CD8/CTL, γδ-T, CAR-T, CAR-NK	Direct target-cell killing and tumor-directed immune activity
Immune-instruction EVs	Dendritic cells, selected γδ-T populations	Antigen presentation, costimulation, adaptive immune priming
Regulatory immune EVs	Treg, selected macrophage states, selected MSCs	Immune suppression or resolution of inflammation
Perinatal regenerative EVs	UC/WJ-MSC, placental MSC, cord-associated sources	Regeneration, immunomodulation, angiogenesis
Adult stromal EVs	BM-MSC, AD-MSC, other MSC sources	Tissue repair and immunomodulation
Neural-lineage EVs	NSC/NPC, neuron, astrocyte, microglia, oligodendrocyte	Neural communication, neuroprotection, or pathological signaling
Cardiovascular EVs	Cardiac progenitor, cardiomyocyte, epicardial, endothelial progenitor	Angiogenesis and myocardial/vascular repair
Pluripotent-cell EVs	ESC, iPSC and derivatives	Developmental/regenerative signaling and engineering platform

Abbreviations: AD-MSC, adipose-derived mesenchymal stromal cell; BM-MSC, bone marrow-derived mesenchymal stromal cell; CAR-NK, chimeric antigen receptor natural killer cell; CAR-T, chimeric antigen receptor T cell; CD8, cluster of differentiation 8; CTL, cytotoxic T lymphocyte; ESC, embryonic stem cell; EV, extracellular vesicle; iPSC, induced pluripotent stem cell; MSC, mesenchymal stromal cell; NK, natural killer cell; NSC/NPC, neural stem/progenitor cell; Treg, regulatory T cell; UC/WJ-MSC, umbilical cord/Wharton’s jelly mesenchymal stromal cell; γδ-T, gamma–delta T cell.

**Table 4:** Representative direct head-to-head EV source comparisons meeting the comparative criteria used in the core evidence layer.

Sources Compared	Context	Main Finding	Interpretation	Ref
Vδ2-T vs. NK vs. DC	EBV-associated tumors	Vδ2-T EVs combined direct cytotoxicity with immune activation and achieved stronger overall tumor control in the tested model.	Relative performance depended on the tested endpoints and tumor model.	[2]
BM-MSC vs. AD-MSC vs. UC-MSC	Wound-healing assays	Relative effects differed between fibroblast and keratinocyte endpoints.	Source effects differed according to the recipient cell and repair endpoint.	[3]
BM-MSC vs. AD-MSC vs. UC-MSC	Proteomic comparison	Distinct protein signatures and pathway enrichments.	Cellular origin shapes cargo architecture.	[4]
BM-MSC vs. AD-MSC vs. UC-MSC	Osteoarthritis models	BMSC and umbilical-MSC EVs were stronger than adipose-derived preparations on several tested endpoints.	The observed ranking was specific to the tested osteoarthritis endpoints.	[7]
Cord blood vs. WJ-MSC vs. placental-MSC sEV	Inflammatory lung-cell models	Viability and cytokine responses differed by source and target cell.	Closely related perinatal sources are not interchangeable.	[8]
Six cardiac/regenerative sources	Cardiac repair	ESC-sEVs showed the strongest overall pro-angiogenic/antifibrotic profile in the tested system.	Tissue origin was not a reliable proxy for potency in the tested system.	[6]

Abbreviations: AD-MSC/ADSC, adipose-derived mesenchymal stromal cell; BM-MSC/BMSC, bone marrow-derived mesenchymal stromal cell; DC, dendritic cell; EBV, Epstein–Barr virus; ESC, embryonic stem cell; EV, extracellular vesicle; MSC, mesenchymal stromal cell; NK, natural killer cell; sEV, small extracellular vesicle; UC-MSC, umbilical cord-derived mesenchymal stromal cell; Vδ2-T, Vδ2 gamma–delta T cell; WJ-MSC, Wharton’s jelly-derived mesenchymal stromal cell.

**Table 5:** Context-dependent advantages and disadvantages of major EV functions.

Property	Potential Advantage	Potential Disadvantage/Contra-Indication
Strong cytotoxicity	Cancer therapy	Neuroregeneration, wound healing, inflammatory disorders
Strong immune activation	Cancer vaccination, selected infections	Autoimmune/inflammatory disease
Immunosuppression	Autoimmune disease, inflammatory injury, transplantation	Cancer surveillance, persistent infection
Angiogenesis	Wound healing, ischemia, tissue regeneration	Selected tumors or pathological neovascularization
Antiapoptotic signaling	Tissue salvage and regeneration	Potential survival support of undesirable cells
High antigen specificity	Antigen-defined cancer	Heterogeneous tumors with antigen loss/escape
Broad innate recognition	Heterogeneous malignancy	Potential off-target effects
Procoagulant surface activity	Potential physiological hemostatic signaling	Systemic IV administration at high dose
Neural trophic signaling	Stroke/neural repair	Limited value when immune cytotoxicity is required

Abbreviations: IV, intravenous.

**Table 6:** Functional identity and best-fit applications of representative EV sources.

Source	Representative Cargo/Identity	Dominant Function	Best-Fit Applications
NK	Perforin, granzymes, granulysin, FasL; activation-dependent phenotype	Direct cytotoxicity	Cancer; engineered targeting
CD8/CTL	Cytotoxic machinery; activation-dependent inflammatory mediators	Antigen-associated cytotoxicity	Cancer
γδ-T/Vδ2-T	FasL, TRAIL, NKG2D, CD80/86, MHC-I/II in selected preparations	Cytotoxicity + immune instruction	Virus-associated and immunogenic tumors
CAR-T	Surface CAR; perforin/granzyme B	Programmable antigen-specific killing	Antigen-defined malignancies
CAR-NK	Engineered CAR + NK cytotoxic program	Programmable cytotoxicity	Cancer
Dendritic cell	Peptide–MHC; costimulatory molecules	Antigen presentation/immune instruction	Cancer vaccination
Treg	Let-7d; miR-150-5p; miR-142-3p	Immune suppression/tolerance	Autoimmune, inflammatory, transplantation
UC/WJ-MSC	miR-181c; broad regenerative and immunoregulatory cargo	Anti-inflammatory + regeneration	Wounds, musculoskeletal, neural injury, inflammation
Placental MSC	Proangiogenic/immunoregulatory cargo; active determinants partly unresolved	Angiogenesis + regeneration	Ischemic/regenerative disease
BM-MSC	Regenerative proteome/RNA cargo	Tissue repair; immunomodulation	Wound, OA, musculoskeletal disease
AD-MSC	Distinct immunoregulatory and regenerative profile	Immunoregulation + repair	Wound/inflammatory applications
NSC/NPC	Neuroregulatory cargo; anti-inflammatory miRNA networks	Neuroprotection/neural repair	Stroke, retinal degeneration, neurodegeneration
Astrocyte/mature neural	State-dependent protective or pathological cargo	Context-dependent neural signaling	Selected neurological disease
Cardiac/epicardial	Cardiac-regenerative miRNAs and proteins	Cardiac repair	Myocardial injury
ESC/iPSC-derived	Developmental/regenerative cargo; engineerable platform	Broad regeneration	Cardiac, neural, engineered delivery

Representative cargo entries indicate molecules reported in association with the corresponding EV preparations and should not be interpreted as established causal mediators unless supported by mechanistic perturbation or direct cargo-manipulation evidence. Mechanistic confidence is evaluated separately using CMEC. Abbreviations: AD-MSC, adipose-derived mesenchymal stromal cell; BM-MSC, bone marrow-derived mesenchymal stromal cell; CAR, chimeric antigen receptor; CAR-NK, chimeric antigen receptor natural killer cell; CAR-T, chimeric antigen receptor T cell; CD8, cluster of differentiation 8; CTL, cytotoxic T lymphocyte; ESC, embryonic stem cell; FasL, Fas ligand; iPSC, induced pluripotent stem cell; MHC, major histocompatibility complex; miR, microRNA; MSC, mesenchymal stromal cell; NKG2D, natural killer group 2 member D; NK, natural killer cell; NSC/NPC, neural stem/progenitor cell; OA, osteoarthritis; RNA, ribonucleic acid; TRAIL, tumor necrosis factor-related apoptosis-inducing ligand; Treg, regulatory T cell; UC/WJ-MSC, umbilical cord/Wharton’s jelly mesenchymal stromal cell; Vδ2-T, Vδ2 gamma–delta T cell; γδ-T, gamma–delta T cell.

**Table 7:** Comparative Evidence Category (CEC) definitions.

Category	Label	Definition
CEC-A	Strong comparative evidence	≥2 independent substantially matched head-to-head comparisons with generally concordant findings, or one substantially matched head-to-head comparison supported by both in vivo confirmation and mechanistic evidence
CEC-B	Moderate comparative evidence	≥1 direct within-study source comparison not fulfilling CEC-A criteria, or ≥2 concordant disease-matched comparative studies
CEC-C	Source-specific evidence	Multiple studies demonstrate biological activity, but robust standardized source-to-source comparisons are lacking
CEC-D	Emerging evidence	Limited or unreplicated proof-of-concept/engineered-platform evidence

Abbreviations: CEC, Comparative Evidence Category.

**Table 8:** EV-FIT+ qualitative source-selection matrix by therapeutic objective.

Therapeutic Objective	Candidate Sources	Principal Strength	Major Trade-Off
Broad tumor cytotoxicity	NK, γδ-T	Natural killing/hybrid immune activity	Specificity, off-target activity, manufacturing
Antigen-specific tumor killing	CAR-T, CAR-NK	Programmable targeting	Antigen escape, engineering complexity
Cancer vaccination/immune instruction	DC, selected γδ-T	Antigen presentation and priming	Limited direct killing, antigen/HLA dependence
Neuroprotection	NSC/NPC, selected MSC sources	Neural support and anti-neuroinflammatory activity	Manufacturing, insufficient head-to-head evidence
Inflammation resolution	UC/WJ, placenta, BM/AD MSC, Treg	Immunoregulation	Excess immunosuppression; context-specific angiogenesis
Wound repair	BM/UC/AD/placental EVs	Regeneration and angiogenesis	Target cell-dependent performance; fibrosis/angiogenesis trade-offs
Cartilage regeneration	BM/UC/synovial MSC candidates	Chondroprotection and inflammation control	Source ranking remains incomplete
Cardiac repair	ESC/iPSC, cardiac lineages, MSCs	Angiogenesis and antifibrotic signaling	Limited clinical evidence; source intuition may mislead

Abbreviations: AD, adipose-derived; BM, bone marrow-derived; CAR-NK, chimeric antigen receptor natural killer cell; CAR-T, chimeric antigen receptor T cell; DC, dendritic cell; ESC, embryonic stem cell; EV, extracellular vesicle; HLA, human leukocyte antigen; iPSC, induced pluripotent stem cell; MSC, mesenchymal stromal cell; NK, natural killer cell; NSC/NPC, neural stem/progenitor cell; Treg, regulatory T cell; UC, umbilical cord; UC/WJ, umbilical cord/Wharton’s jelly; γδ-T, gamma–delta T cell.

**Table 9:** Minimum recommended dataset for future head-to-head EV studies.

Domain	Minimum Recommended Reporting
Parent-cell variables	Exact cell type/subtype; tissue/anatomic source; donor number; donor age where relevant; passage; culture medium; oxygen; activation/preconditioning; confluence; parental-cell engineering status; and, where applicable, post-isolation EV engineering reported separately.
EV preparation	Isolation/purification method; size distribution; particle concentration; total protein; EV-associated markers; contaminant assessment; morphology; small vs. medium/large fraction.
Functional variables	Mechanism-linked potency assay; target cell; dose–response; relevant cargo/pathway; contra-function testing; matched comparator sources.
Translational variables	Biodistribution; route; repeat dosing; immunogenicity; coagulation; cytokine release; off-target toxicity; storage; manufacturing reproducibility; batch-to-batch consistency; scalability; and evidence of therapeutic efficacy.

Abbreviations: EV, extracellular vesicle.

## Data Availability

No new data were created or analyzed in this study. Data sharing is not applicable to this article.

## Supplementary Materials

The following supporting information can be downloaded at: https://www.mdpi.com/article/10.3390/cells15191731/s1. Table S1: Full search strategies and search dates; Table S2: Numerical search, deduplication, and screening summary; Table S3: Eligibility and screening framework; Table S4: Complete direct source-comparison study inventory (n = 70); Table S5: Study-level methodological reporting appraisal.

## Abbreviations

AD-MSC, adipose-derived mesenchymal stromal cell; BM-MSC, bone marrow-derived mesenchymal stromal cell; DC, dendritic cell; ESC, embryonic stem cell; EV, extracellular vesicle; iPSC, induced pluripotent stem cell; MSC, mesenchymal stromal cell; NK, natural killer cell; NSC/NPC, neural stem/progenitor cell; Treg, regulatory T cell; UC/WJ-MSC, umbilical cord/Wharton’s jelly mesenchymal stromal cell; γδ-T, gamma–delta T cell.
