# Supplementary material for: Not All Extracellular Vesicles Are Equal: A Narrative Comparative Review of Source-Dependent Function and Therapeutic Potential
Source: Cells. 2026 Sep 23;15(19):1731. doi: 10.3390/cells15191731 (PMC13664309; doi:10.3390/cells15191731)
Supplement: Supplementary file 1 [file cells-15-01731-s001.zip › cells-4545327-supplementary.pdf]

Not All Extracellular Vesicles Are Equal: A Narrative Comparative Review of Source-Dependent Function and Therapeutic Potential

Supplementary Materials

Supplementary Table S1. Full search strategies and search dates (Final search date: 14 August 2026)

Source	Coverage / date	Records identified	Exact strategy / role
PubMed/MEDLINE	Inception–14 Aug 2026	5,527	(<br>"extracellular vesicle*"[Title/Abstract]<br>OR exosome*[Title/Abstract]<br>OR "small extracellular vesicle*"[Title/Abstract]<br>OR sEV[Title/Abstract]<br>OR sEVs[Title/Abstract]<br>)<br>AND<br>(<br>"natural killer"[Title/Abstract] OR NK[Title/Abstract] OR "T cell*"[Title/Abstract]<br>OR "T-cell*"[Title/Abstract] OR "gamma delta T"[Title/Abstract] OR "γδ T"[Title/Abstract]<br>OR Vdelta2[Title/Abstract] OR "CAR-T"[Title/Abstract] OR "CAR NK"[Title/Abstract]<br>OR dendritic[Title/Abstract] OR Treg[Title/Abstract]<br>OR "mesenchymal stem cell*"[Title/Abstract] OR "mesenchymal stromal cell*"[Title/Abstract]<br>OR MSC[Title/Abstract] OR "umbilical cord"[Title/Abstract] OR "Wharton* jelly"[Title/Abstract]<br>OR placenta*[Title/Abstract] OR "bone marrow"[Title/Abstract] OR adipose[Title/Abstract]<br>OR "neural stem"[Title/Abstract] OR "neural progenitor"[Title/Abstract]<br>OR neuron*[Title/Abstract] OR astrocyte*[Title/Abstract] OR microglia*[Title/Abstract]<br>OR oligodendrocyte*[Title/Abstract] OR "cardiac progenitor"[Title/Abstract]<br>OR cardiomyocyte*[Title/Abstract] OR epicardial[Title/Abstract] OR "endothelial progenitor"[Title/Abstract]<br>OR "embryonic stem"[Title/Abstract] OR "induced pluripotent"[Title/Abstract] OR iPSC[Title/Abstract]<br>)<br>AND<br>(<br>compar*[Title/Abstract] OR "head-to-head"[Title/Abstract] OR "different source*"[Title/Abstract]<br>OR "different cell source*"[Title/Abstract] OR "source-dependent"[Title/Abstract]<br>OR "source specific"[Title/Abstract] OR "source-specific"[Title/Abstract]<br>OR "cellular origin"[Title/Abstract] OR "tissue origin"[Title/Abstract]<br>)
Europe PMC	Inception–14 Aug 2026	3,633	TITLE_ABS:(<br>("extracellular vesicle*" OR exosome* OR "small extracellular vesicle*" OR sEV OR sEVs)<br>AND<br>("natural killer" OR NK OR "T cell*" OR "T-cell*" OR "gamma delta T" OR Vdelta2<br>OR "CAR-T" OR "CAR NK" OR dendritic OR Treg<br>OR "mesenchymal stem cell*" OR "mesenchymal stromal cell*" OR MSC<br>OR "umbilical cord" OR "Wharton* jelly" OR placenta* OR "bone marrow" OR adipose<br>OR "neural stem" OR "neural progenitor" OR neuron* OR astrocyte* OR microglia* OR oligodendrocyte*<br>OR "cardiac progenitor" OR cardiomyocyte* OR epicardial OR "endothelial progenitor"<br>OR "embryonic stem" OR "induced pluripotent" OR iPSC)<br>AND<br>(compar* OR "head-to-head" OR "different source*" OR "different cell source*"<br>OR "source-dependent" OR "source specific" OR "source-specific" OR "cellular origin" OR "tissue origin")<br>)
OpenAlex (works; title/abstract search)	Inception–14 Aug 2026	619	("extracellular vesicles" OR exosomes) AND<br>(comparative OR "head-to-head" OR "source-dependent" OR "different sources") AND<br>(MSC OR "natural killer" OR dendritic OR neural OR cardiac OR placenta OR "umbilical cord" OR adipose OR "bone marrow" OR iPSC)
Google Scholar / citation searching	14 Aug 2026	Not used as a raw database count	Targeted supplementary searching only; six prespecified targeted queries plus backward/forward citation searching. Three unique eligible studies were added after deduplication against the formal database set.

Google Scholar targeted queries: "extracellular vesicles" "different sources" comparative \| exosomes "different sources" mesenchymal stem cells comparative \| exosomes "bone marrow" adipose "umbilical cord" comparison \| "extracellular vesicles" NK dendritic T cells comparison \| "extracellular vesicles" neural cardiac placenta

comparative | "extracellular vesicles" "head-to-head" source. Approximate Google Scholar hit counts were not used in the PRISMA numerical database flow because they are unstable and ranking-dependent.

Supplementary Table S2. Numerical search, deduplication, and screening summary

PRISMA stage	Item	n
Identification—databases	PubMed/MEDLINE records identified	5,527
Identification—databases	Europe PMC records identified	3,633
Identification—databases	OpenAlex records identified	619
Identification—databases	Total database records	9,779
Before screening	Duplicate records removed	3,799
Screening	Unique records screened by title/abstract	5,980
Screening	Records excluded for not meeting direct source-comparison criteria	5,913
Eligibility—databases	Reports sought and assessed	67
Eligibility—databases	Reports not retrieved	0
Eligibility—databases	Reports excluded after final eligibility verification	0
Other methods	Unique reports identified by targeted Google Scholar / citation searching	3
Other methods	Reports sought and assessed	67
Other methods	Reports excluded	0
Included	Direct source-comparison studies included in systematic layer	70

Deduplication used DOI and PMID identifiers supplemented by normalized title/author matching. The primary systematic layer required an explicit within-study comparison of EV preparations from at least two distinct parental cell or tissue sources. Contextual single-source studies used elsewhere in the narrative review were not counted in the PRISMA included-study total.

Supplementary Table S3. Eligibility and screening framework

Domain	Include / approach	Exclude / limitation
Study design	Original peer-reviewed mammalian research; in vitro, ex vivo, animal, or human/translational.	Reviews/meta-analyses, editorials, protocols, conference-only records, preprints.
Comparison unit	≥2 source-defined EV/sEV/exosome preparations compared within the same study.	Single-source EV studies; EV-vs-parent-cell only; same-source conditioning/preconditioning-only comparisons.
EV identity	Distinct parental cell/tissue sources with separable EV data.	Non-EV secretome without separable EV data; source not identifiable; microbial/plant vesicles.
Eligible endpoints	Cargo/surface phenotype, uptake, mechanism, potency, therapeutic function, safety/contra-function, yield/manufacturing, translational performance.	Biomarker-only or diagnostic cell-of-origin studies without source-to-function comparison.
Search stage	No year or open-access restriction applied at search stage.	Records after 14 Aug 2026.
Synthesis	Narrative comparative synthesis because of major design/outcome heterogeneity.	No pooled meta-analysis or universal source-ranking effect size.
Risk/quality handling	Descriptive methodological reporting domains in Table S5; not a validated pooled risk-of-bias score.	No unsupported cross-design numerical risk score.

Supplementary Table S4. Complete direct source-comparison study inventory (n = 70)

#	Year	EV sources compared	Primary comparative focus	Study title	Identifier	Discovery
1	2015	MSC sources (including BM/AD/UC)	Glioblastoma-cell response	Differential effects of extracellular vesicles secreted by mesenchymal stem cells from different sources on glioblastoma cells	PMID 25539575; DOI 10.1517/14712598.2015.997706	Formal database search
2	2015	BM-MSC vs AD-MSC	RNA cargo profiling	Human bone marrow- and adipose-mesenchymal stem cells secrete exosomes enriched in distinctive miRNA and tRNA species.	PMID 26129847; DOI 10.1186/s13287-015-0116-z	Formal database search
3	2016	Normal T-cell blast vs Jurkat T-cell line	Proteomic cargo and tumorigenic signaling	Comparative proteomics of exosomes secreted by tumoral Jurkat T cells and normal human T cell blasts unravels a potential tumorigenic role for valosin-containing protein	PMID 27086912; DOI 10.18632/oncotarget.8678	Formal database search
4	2016	U87 glioblastoma vs Huh7 hepatocellular carcinoma vs BM-MSC	Proteomic and lipidomic cargo	High-resolution proteomic and lipidomic analysis of exosomes and microvesicles from different cell sources	PMID 27863537; DOI 10.3402/jev.v5.32570	Formal database search
5	2016	MSC sources	Neuritic outgrowth	Mesenchymal stem cell-derived exosomes from different sources selectively promote neuritic outgrowth.	PMID 26851773; DOI 10.1016/j.neuroscience.2016.01.061	Formal database search
6	2017	iPSC-MSC vs synovial MSC	Osteoarthritis	Comparison of exosomes secreted by induced pluripotent stem cell-derived mesenchymal stem cells and synovial membrane-derived mesenchymal stem cells for the treatment of osteoarthritis	PMID 28279188; DOI 10.1186/s13287-017-0510-9	Formal database search
7	2017	hESC-CM vs hiPSC-CM	Non-coding RNA cargo and cardiac efficacy	Comparison of Non-Coding RNAs in Exosomes and Functional Efficacy of Human Embryonic Stem Cell- versus Induced Pluripotent Stem Cell-Derived Cardiomyocytes.	PMID 28710827; DOI 10.1002/stem.2669	Formal database search
8	2019	BM-MSC vs amniotic-fluid MSC	Yield and EV characteristics	A comparison of clinically relevant sources of mesenchymal stem cell-derived exosomes: Bone marrow and amniotic fluid.	PMID 30361074; DOI 10.1016/j.jpedsurg.2018.10.020	Formal database search
9	2019	AD-MSC vs BM-MSC	Secretome/EV characterization and immunomodulation	Comparative analysis and characterization of soluble factors and exosomes from cultured adipose tissue and bone marrow mesenchymal stem cells in canine species.	PMID 30712794; DOI 10.1016/j.vetimm.2018.12.003	Formal database search
10	2019	Endothelial cell vs endothelial progenitor cell	Vascular endothelial repair	Comparison of endothelial cell- and endothelial progenitor cell-derived exosomes in promoting vascular endothelial cell repair	PMID 31934115	Formal database search
11	2019	BM-MSC vs dental-pulp stem cell	Immunomodulation	Comparison of immunomodulatory properties of exosomes derived from bone marrow mesenchymal stem cells and dental pulp stem cells.	PMID 31407157; DOI 10.1007/s12026-019-09088-6	Formal database search
12	2020	BM-MSC vs AD-MSC vs UC-MSC	Proteomic cargo	Comprehensive proteomic analysis of exosomes derived from human bone marrow, adipose tissue, and umbilical cord mesenchymal stem cells.	PMID 33246507; DOI 10.1186/s13287-020-02032-8	Targeted Google Scholar / citation searching
13	2020	BM-MSC vs AD-MSC vs UC-MSC	Wound-healing endpoints	Differential wound healing capacity of mesenchymal stem cell-derived exosomes originated from bone marrow, adipose tissue and umbilical cord under serum- and xeno-free condition.	PMID 32671095; DOI 10.3389/fmolb.2020.00119	Targeted Google Scholar / citation searching
14	2020	Vδ2-T vs NK vs dendritic-cell exosomes	Antitumor cytotoxicity and immune activation	Exosomes derived from Vδ2-T cells control Epstein-Barr virus-associated tumors and induce T cell antitumor immunity.	PMID 32998970; DOI 10.1126/scitranslmed.aaz3426	Formal database search
15	2020	Three stem/progenitor cell types	Hyperglycemia-dependent EV transcriptome	Identification and Comparison of Hyperglycemia-Induced Extracellular Vesicle Transcriptome in Different Mouse Stem Cells	PMID 32942572; DOI 10.3390/cells9092098	Formal database search
16	2020	Apical-papilla stem cell vs BM-MSC	piRNA cargo	Identification and Comparison of piRNA Expression Profiles of Exosomes Derived from Human Stem Cells from the Apical Papilla and Bone Marrow Mesenchymal Stem Cells	PMID 32031053; DOI 10.1089/scd.2019.0277	Formal database search
17	2021	AD-MSC vs BM-MSC	Osteoarthritis	A Comparative Study on the Effect of Exosomes Secreted by Mesenchymal Stem Cells Derived from Adipose and Bone Marrow Tissues in the Treatment of Osteoarthritis-Induced Mouse Model	PMID 34616850; DOI 10.1155/2021/9688138	Formal database search
18	2021	Skeletal muscle vs adipose tissue	miRNA cargo and adipogenesis	Comparative Analysis of MicroRNA Expression Profiles Between Skeletal Muscle- and Adipose-Derived Exosomes in Pig	PMID 34135937; DOI 10.3389/fgene.2021.631230	Formal database search
19	2021	AD-MSC vs amniotic-membrane MSC	miRNA cargo in osteoarthritis	Comparison of miRNA cargo in human adipose-tissue vs. amniotic-membrane derived mesenchymal stromal cells extracellular vesicles for osteoarthritis treatment	PMID 39697592; DOI 10.20517/evcna.2021.11	Formal database search
20	2021	BM-MSC vs AD-MSC	Diabetic wound healing and cargo	Differential Therapeutic Effect of Extracellular Vesicles Derived by Bone Marrow and Adipose Mesenchymal Stem	PMID 33917759; DOI 10.3390/ijms22083851	Formal database search

#	Year	EV sources compared	Primary comparative focus	Study title	Identifier	Discovery
				Cells on Wound Healing of Diabetic Ulcers and Correlation to Their Cargoes		
21	2021	Three MSC tissue sources	miRNA cargo, immune function, neurogenesis	MicroRNA-Enriched Exosomes from Different Sources of Mesenchymal Stem Cells Can Differentially Modulate Functions of Immune Cells and Neurogenesis	PMID 35052749; DOI 10.3390/biomedicines10010069	Formal database search
22	2021	UC-MSC vs AD-MSC	Proteomic/functional differences	Proteomics Analyses Reveal Functional Differences between Exosomes of Mesenchymal Stem Cells Derived from The Umbilical Cord and Those Derived from The Adipose Tissue.	PMID 33650823; DOI 10.22074/cellj.2021.6969	Targeted Google Scholar / citation searching
23	2021	AD-MSC vs BM-MSC	Endothelial proliferation and chondrogenesis	Role of extracellular vesicles from adipose tissue- and bone marrow-mesenchymal stromal cells in endothelial proliferation and chondrogenesis.	PMID 34480533; DOI 10.1002/sctm.21-0107	Formal database search
24	2021	BM-MSC vs AD-MSC	Cartilage and bone regeneration	The tissue origin effect of extracellular vesicles on cartilage and bone regeneration.	PMID 33657452; DOI 10.1016/j.actbio.2021.02.039	Formal database search
25	2021	BM-MSC vs AD-MSC	Tissue-regeneration capacity	Tissue Regeneration Capacity of Extracellular Vesicles Isolated From Bone Marrow-Derived and Adipose-Derived Mesenchymal Stromal/Stem Cells	PMID 33718390; DOI 10.3389/fcell.2021.648098	Formal database search
26	2022	iPSC vs tissue-specific MSC sources	Proteomic cargo	Comparative proteomic profiling of Small Extracellular vesicles derived from iPSCs and tissue specific mesenchymal stem cells	PMID 36126717; DOI 10.1016/j.yexcr.2022.113354	Formal database search
27	2022	Primary NK vs NK-92 vs KHYG-1	Tumor targeting and cytolytic proteins	Comparison of characteristics and tumor targeting properties of extracellular vesicles derived from primary NK cells or NK-cell lines stimulated with IL-15 or IL-12/15/18	PMID 35119498; DOI 10.1007/s00262-022-03161-0	Formal database search
28	2022	AD-MSC vs BM-MSC vs UC-MSC	Sepsis-induced acute lung injury	Differential Lung Protective Capacity of Exosomes Derived from Human Adipose Tissue, Bone Marrow, and Umbilical Cord Mesenchymal Stem Cells in Sepsis-Induced Acute Lung Injury.	PMID 35265265; DOI 10.1155/2022/7837837	Formal database search
29	2022	hESC vs hiPSC vs UC-MSC	Proteomic and miRNA cargo	Systemic proteomics and miRNA profile analysis of exosomes derived from human pluripotent stem cells.	PMID 36064647; DOI 10.1186/s13287-022-03142-1	Formal database search
30	2023	Platelet vs UC-MSC	Ex vivo inflammatory osteoarthritis	Comparative effect of platelet- and mesenchymal stromal cell-derived extracellular vesicles on human cartilage explants using an ex vivo inflammatory osteoarthritis model	PMID 37852621; DOI 10.1302/2046-3758.1210.BJR-2023-0109.R1	Formal database search
31	2023	BM-MSC vs brain endothelial cell	BBB protection in ischemic stroke	Comparative study of extracellular vesicles derived from mesenchymal stem cells and brain endothelial cells attenuating blood-brain barrier permeability via regulating Caveolin-1-dependent ZO-1 and Claudin-5 endocytosis in acute ischemic stroke	PMID 36855156; DOI 10.1186/s12951-023-01828-z	Formal database search
32	2023	Melanoma/anaplastic cell vs iPSC	Metastasis suppression	Comparative Study of Metastasis Suppression Effects of Extracellular Vesicles Derived from Anaplastic Cell Lines, Nanog-Overexpressing Melanoma, and Induced Pluripotent Stem Cells	PMID 38139035; DOI 10.3390/ijms242417206	Formal database search
33	2023	Synovial-fluid MSC vs AD-MSC	miRNA-127-5p and chondrogenesis	Comparison of exosomes secreted by synovial fluid-derived mesenchymal stem cells and adipose tissue-derived mesenchymal stem cells in culture for microRNA-127-5p expression during chondrogenic differentiation	PMID 36878417; DOI 10.1016/j.gene.2023.147337	Formal database search
34	2023	PBMC/fibroblast/urine cell vs derived iPSC	Exosomal miRNA transcriptome	Comparison of miRNA transcriptome of exosomes in three categories of somatic cells with derived iPSCs	PMID 37696871; DOI 10.1038/s41597-023-02493-5	Formal database search
35	2023	Source-defined exosomes	Tendon-repair markers	Exosome Cell Origin Affects In Vitro Markers of Tendon Repair in Ovine Macrophages and Tenocytes.	PMID 36792933; DOI 10.1089/ten.tea.2022.0185	Formal database search
36	2023	Multiple MSC sources	Experimental sepsis	Extracellular Vesicles from Different Sources of Mesenchymal Stromal Cells Have Distinct Effects on Lung and Distal Organs in Experimental Sepsis	PMID 37175936; DOI 10.3390/ijms24098234	Formal database search
37	2024	CD8 αβ T vs γδ T	Proteomic and cytotoxic phenotype	Comparative Analysis of Extracellular Vesicles from Cytotoxic CD8(+) αβ T Cells and γδ T Cells	PMID 39451262; DOI 10.3390/cells13201745	Formal database search
38	2024	BM-MSC vs WJ-MSC	EV morphology, protein and size	Comparison of morphology, protein concentration, and size distribution of bone marrow and Wharton's jelly-derived mesenchymal stem cells exosomes isolated by ultracentrifugation and polymer-based precipitation	PMID 38833940; DOI 10.1016/j.tice.2024.102427	Formal database search
39	2024	Feline AD-MSC vs placental stromal cell	EV phenotype and regenerative potential	Distinctive characteristics of extracellular vesicles from feline adipose and placenta stromal cells unveil potential for regenerative medicine in cats	PMID 38479108; DOI 10.2460/javma.23.11.0662	Formal database search
40	2024	BM-MSC vs AD-MSC	Bone-tendon healing	Effect of Exosomes From Bone Marrow-Derived Mesenchymal Stromal Cells and Adipose-Derived Stromal	PMID 38188618; DOI 10.1177/23259671231210304	Formal database search

#	Year	EV sources compared	Primary comparative focus	Study title	Identifier	Discovery
				Cells on Bone-Tendon Healing in a Murine Rotator Cuff Injury Model.		
41	2024	iPSC-MSC vs BM-MSC	Doxorubicin cardiomyopathy	Exosomal miR-9-5p derived from iPSC-MSCs ameliorates doxorubicin-induced cardiomyopathy by inhibiting cardiomyocyte senescence.	PMID 38643173; DOI 10.1186/s12951-024-02421-8	Formal database search
42	2024	Six regenerative/cardiac sources	Cardiac repair	Head-to-head comparison of relevant cell sources of small extracellular vesicles for cardiac repair: Superiority of embryonic stem cells.	PMID 38711334; DOI 10.1002/jev2.12445	Formal database search
43	2024	Nucleus-pulposus cell vs MSC	Disc degeneration and pain	ISSLS PRIZE in Basic Science 2024: superiority of nucleus pulposus cell- versus mesenchymal stromal cell-derived extracellular vesicles in attenuating disc degeneration and alleviating pain	PMID 38416190; DOI 10.1007/s00586-024-08163-3	Formal database search
44	2024	BM-MSC vs corneal epithelial cell	Corneal repair, apoptosis, immunomodulation	Mesenchymal vs. epithelial extracellular vesicles in corneal epithelial repair, apoptosis, and immunomodulation: An in vitro study	PMID 39127238; DOI 10.1016/j.exer.2024.110027	Formal database search
45	2024	CD8 T cell vs cancer-associated fibroblast	Opposing lung-cancer effects	Research on the mechanism of exosomes from different sources influencing the progression of lung cancer	PMID 38760988; DOI 10.1002/tox.24292	Formal database search
46	2024	Cord-blood/perinatal vs placental MSC sEVs	Inflammatory lung injury	Small Extracellular Vesicles Derived from Cord Blood Plasma and Placental Mesenchymal Stem Cells Attenuate Acute Lung Injury Induced by Lipopolysaccharide (LPS).	PMID 39795932; DOI 10.3390/ijms26010075	Formal database search
47	2024	Cardiac progenitor cell vs MSC membranes	sEV-like vehicle composition and function	The Effect of Parent Cell Type on Small Extracellular Vesicle-Derived Vehicle Functionality.	PMID 38143286; DOI 10.1002/adbi.202300462	Formal database search
48	2024	Schwann cell vs fibroblast vs neural stem cell	Schwann-cell differentiation	The effects of exosomes originating from different cell sources on the differentiation of bone marrow mesenchymal stem cells into schwann cells	PMID 38698449; DOI 10.1186/s12951-024-02450-3	Formal database search
49	2024	Engineered RAW264.7 macrophage vs placental MSC	Sepsis-induced acute lung injury	Therapeutic Effects of Engineered Exosomes from RAW264.7 Cells Overexpressing hsa-let-7i-5p against Sepsis in Mice-A Comparative Study with Human Placenta-Derived Mesenchymal Stem Cell Exosomes	PMID 38929840; DOI 10.3390/jpm14060619	Formal database search
50	2024	Fetal-liver MSC vs adult BM-MSC	Transcriptomic/proteomic EV differences	Transcriptomic and proteomic profiles of fetal versus adult mesenchymal stromal cells and mesenchymal stromal cell-derived extracellular vesicles	PMID 38475970; DOI 10.1186/s13287-024-03683-7	Formal database search
51	2025	iPSC-derived MSC vs AD-MSC	Regenerative bioactivity	Comparative analysis of extracellular vesicles from induced and adipose-derived Mesenchymal Stem Cells: Implications for regenerative medicine	PMID 40465768; DOI 10.1371/journal.pone.0325065	Formal database search
52	2025	BM-MSC vs AD-MSC vs umbilical MSC	Osteoarthritis	Comparative Efficacy of Exosomes Derived from Different Mesenchymal Stem Cell Sources in Osteoarthritis Models: An In Vitro and Ex Vivo Analysis.	PMID 40564912; DOI 10.3390/ijms26125447	Formal database search
53	2025	Glioblastoma vs neuron/astrocyte/brain endothelial cell	Proteomic cargo and cancer-associated proteins	Comparative proteomic profiling of glioblastoma and healthy brain cell-derived extracellular vesicles reveals enrichment of cancer-associated proteins	PMID 40058457; DOI 10.1016/j.jprot.2025.105418	Formal database search
54	2025	BM-MSC vs cardiomyocyte	Ischemic cardiomyocyte injury	Comparative study the effect of human bone marrow mesenchymal stem cells and cardiomyocytes derived exosomes on improvement of Isoproterenol induced ischemic damaged cardiomyocytes	PMID 40756127; DOI 10.46582/jsrm.2101004	Formal database search
55	2025	iPSC vs AD-MSC vs BM-MSC	miRNA cargo and chondrocyte function	Comparisons of miRNA profiles of exosomes derived from human iPSCs, ADSCs, and BMSCs and effects on chondrocyte function.	PMID 40823751; DOI 10.1302/2046-3758.148.bjr-2024-0267.r4	Formal database search
56	2025	Induced MSC vs BM-MSC	Anticancer activity	Distinct anticancer properties of exosomes from induced mesenchymal stem cells vs. bone marrow-derived stem cells in MCF7 and A549 models.	PMID 40420975; DOI 10.3892/br.2025.1994	Formal database search
57	2025	AD-MSC vs UC-MSC	Psoriasis	Exosome-Based Therapy: A Comparative Study of Adipose- and Umbilical Cord-Derived Mesenchymal Stem Cells-Derived Exosomes in Psoriatic Mouse Model.	PMID 41217063; DOI 10.1111/exd.70176	Formal database search
58	2025	Placental, endometrial and dental-pulp MSC types	Surface proteins and molecular phenotype	Extracellular Vesicles from Different Mesenchymal Stem Cell Types Exhibit Distinctive Surface Protein Profiling and Molecular Characteristics: A Comparative Analysis	PMID 40244251; DOI 10.3390/ijms26073393	Formal database search
59	2025	iPSC-cardiomyocyte vs MSC	Porcine myocardial injury	Molecular Mechanisms of Exosomes From Human iPSC-Cardiomyocytes and Mesenchymal Stem Cells in Restoring the Injured Myocardium.	PMID 41128145; DOI 10.1161/jaha.124.037005	Formal database search
60	2025	Neural progenitor vs developing neuron	Microglial activation	Neural Progenitor Cell- and Developing Neuron-Derived Extracellular Vesicles Differentially Modulate Microglial Activation	PMID 40806231; DOI 10.3390/ijms26157099	Formal database search

#	Year	EV sources compared	Primary comparative focus	Study title	Identifier	Discovery
61	2025	Tenocyte vs MSC exosomes	Tendon-healing phenotypes	Primary Human Macrophage and Tenocyte Tendon Healing Phenotypes Changed by Exosomes Per Cell Origin.	PMID 39761039; DOI 10.1089/ten.tea.2024.0143	Formal database search
62	2026	UC-MSC vs AD-MSC	Human skin regeneration	Biologically Distinct, Clinically Convergent: A Comparative Study of Umbilical Cord- and Adipose-Derived Mesenchymal Stem Cell Exosomes in Human Skin Regeneration.	PMID 41800728; DOI 10.1093/asj/sjaf254	Formal database search
63	2026	Canine placental MSC vs AD-MSC	Immunomodulation	Comparative analysis of the immunomodulatory effects of extracellular vesicles from canine placental and adipose-derived mesenchymal stem cells	PMID 41702062; DOI 10.2460/ajvr.25.12.0426	Formal database search
64	2026	iPSC-microglia vs neural stem cell	Alzheimer model / cognitive decline	Comparing Functional Consequences of Human iPSC-Microglia and Neural Stem Cell-Derived Extracellular Vesicles in Mitigating Cognitive Decline in Alzheimer's Disease	PMID 41432310; DOI 10.1111/acel.70341	Formal database search
65	2026	MSC vs NK-cell EVs	Endometrial-cancer cisplatin responsiveness	Differential Effects of Mesenchymal Stem Cell- and Natural Killer Cell-Derived Extracellular Vesicles on Cisplatin Responsiveness in Endometrial Cancer Cells	PMID 42450114; DOI 10.3390/ijms27135842	Formal database search
66	2026	Chondroprogenitor vs chondrocyte vs BM-MSC	Repair and immunomodulation	Distinct Reparative and Immunomodulatory Signatures of Extracellular Vesicles From Human Cartilage-Derived Chondroprogenitors, Chondrocytes, and Bone Marrow Mesenchymal Stem Cells	PMID 42541371; DOI 10.1177/19476035261475567	Formal database search
67	2026	iPSC-MSC vs UC-MSC	Cisplatin-induced ovarian toxicity	Exosomes derived from different sources of mesenchymal stem cells attenuate cisplatin-induced ovarian toxicity	PMID 42546485; DOI 10.1016/j.jri.2026.104942	Formal database search
68	2026	BM-MSC vs AD-MSC	Bone regeneration	Extracellular Vesicles (EVs) Mirror Their Source: Comparative Analysis of Bone Marrow-Derived and Adipose-Derived Stem Cells and EVs in Bone Regeneration	PMID 42454286; DOI 10.1007/s12195-026-00909-x	Formal database search
69	2026	UC-MSC vs AD-MSC	Postoperative intra-abdominal adhesions	Mesenchymal stem cells and their exosomes from umbilical cord and adipose tissue in the prevention of postoperative intra-abdominal adhesions: A rat model study.	PMID 41997426; DOI 10.1016/j.biocel.2026.106948	Formal database search
70	2026	Six source-defined cell types	Cell survival and neuritogenesis	Small extracellular vesicles promote cell survival and neuritogenesis in vitro in a manner dependent on dosage and cell of origin.	PMID 42236787; DOI 10.1038/s41598-026-53763-2	Formal database search

Supplementary Table S5. Study-level methodological reporting appraisal

This descriptive appraisal records design features visible from the exported title/abstract/metadata and is not presented as a validated risk-of-bias instrument. “NR” means not clearly reported in the exported abstract/metadata; it does not imply that the information is absent from the full article.

#	Year	Sources	Within-study matched source comparison	EV characterization visible	Dose normalization visible	Functional endpoint	In vivo/ex vivo component	Cargo/mechanistic linkage
1	2015	MSC sources (including BM/AD/UC)	Yes	NR	NR	No/omics-focused	NR	NR
2	2015	BM-MSC vs AD-MSC	Yes	Yes	NR	No/omics-focused	NR	Yes
3	2016	Normal T-cell blast vs Jurkat T-cell line	Yes	Yes	NR	Yes	Yes	Yes
4	2016	U87 glioblastoma vs Huh7 hepatocellular carcinoma vs BM-MSC	Yes	Yes	NR	No/omics-focused	NR	Yes
5	2016	MSC sources	Yes	NR	NR	No/omics-focused	NR	NR
6	2017	iPSC-MSC vs synovial MSC	Yes	NR	NR	Yes	Yes	NR
7	2017	hESC-CM vs hiPSC-CM	Yes	Yes	NR	No/omics-focused	NR	Yes
8	2019	BM-MSC vs amniotic-fluid MSC	Yes	Yes	NR	No/omics-focused	NR	NR
9	2019	AD-MSC vs BM-MSC	Yes	Yes	NR	Yes	Yes	NR
10	2019	Endothelial cell vs endothelial progenitor cell	Yes	NR	NR	Yes	NR	NR
11	2019	BM-MSC vs dental-pulp stem cell	Yes	NR	NR	Yes	NR	NR
12	2020	BM-MSC vs AD-MSC vs UC-MSC	Yes	Yes	NR	No/omics-focused	NR	Yes
13	2020	BM-MSC vs AD-MSC vs UC-MSC	Yes	NR	NR	Yes	NR	NR
14	2020	Vδ2-T vs NK vs dendritic-cell exosomes	Yes	NR	NR	Yes	NR	NR
15	2020	Three stem/progenitor cell types	Yes	Yes	NR	No/omics-focused	Yes	Yes
16	2020	Apical-papilla stem cell vs BM-MSC	Yes	Yes	NR	No/omics-focused	NR	Yes
17	2021	AD-MSC vs BM-MSC	Yes	NR	NR	Yes	Yes	NR
18	2021	Skeletal muscle vs adipose tissue	Yes	Yes	NR	Yes	Yes	Yes
19	2021	AD-MSC vs amniotic-membrane MSC	Yes	Yes	NR	Yes	Yes	Yes
20	2021	BM-MSC vs AD-MSC	Yes	Yes	NR	Yes	NR	Yes
21	2021	Three MSC tissue sources	Yes	Yes	NR	Yes	NR	Yes
22	2021	UC-MSC vs AD-MSC	Yes	Yes	NR	No/omics-focused	NR	Yes
23	2021	AD-MSC vs BM-MSC	Yes	NR	NR	No/omics-focused	Yes	NR
24	2021	BM-MSC vs AD-MSC	Yes	NR	NR	Yes	Yes	NR
25	2021	BM-MSC vs AD-MSC	Yes	NR	NR	Yes	Yes	NR
26	2022	iPSC vs tissue-specific MSC sources	Yes	Yes	NR	No/omics-focused	Yes	Yes
27	2022	Primary NK vs NK-92 vs KHYG-1	Yes	Yes	NR	Yes	NR	NR
28	2022	AD-MSC vs BM-MSC vs UC-MSC	Yes	NR	NR	Yes	Yes	NR
29	2022	hESC vs hiPSC vs UC-MSC	Yes	Yes	NR	No/omics-focused	NR	Yes
30	2023	Platelet vs UC-MSC	Yes	NR	NR	Yes	Yes	NR
31	2023	BM-MSC vs brain endothelial cell	Yes	NR	NR	Yes	Yes	NR
32	2023	Melanoma/anaplastic cell vs iPSC	Yes	NR	NR	Yes	Yes	NR
33	2023	Synovial-fluid MSC vs AD-MSC	Yes	Yes	NR	Yes	NR	Yes
34	2023	PBMC/fibroblast/urine cell vs	Yes	Yes	NR	No/omics-focused	NR	Yes

#	Year	Sources	Within-study matched source comparison	EV characterization visible	Dose normalization visible	Functional endpoint	In vivo/ex vivo component	Cargo/mechanistic linkage
		derived iPSC						
35	2023	Source-defined exosomes	Yes	NR	NR	Yes	NR	NR
36	2023	Multiple MSC sources	Yes	NR	NR	Yes	Yes	NR
37	2024	CD8 αβ T vs γδ T	Yes	Yes	NR	Yes	Yes	Yes
38	2024	BM-MSC vs WJ-MSC	Yes	Yes	NR	No/omics-focused	Yes	NR
39	2024	Feline AD-MSC vs placental stromal cell	Yes	Yes	NR	Yes	Yes	NR
40	2024	BM-MSC vs AD-MSC	Yes	NR	NR	Yes	NR	NR
41	2024	iPSC-MSC vs BM-MSC	Yes	NR	NR	No/omics-focused	Yes	NR
42	2024	Six regenerative/cardiac sources	Yes	NR	NR	Yes	NR	NR
43	2024	Nucleus-pulposus cell vs MSC	Yes	NR	NR	No/omics-focused	Yes	NR
44	2024	BM-MSC vs corneal epithelial cell	Yes	NR	NR	Yes	NR	NR
45	2024	CD8 T cell vs cancer-associated fibroblast	Yes	NR	NR	Yes	NR	NR
46	2024	Cord-blood/perinatal vs placental MSC sEVs	Yes	NR	NR	No/omics-focused	NR	NR
47	2024	Cardiac progenitor cell vs MSC membranes	Yes	NR	NR	No/omics-focused	NR	NR
48	2024	Schwann cell vs fibroblast vs neural stem cell	Yes	NR	NR	Yes	NR	NR
49	2024	Engineered RAW264.7 macrophage vs placental MSC	Yes	NR	NR	Yes	Yes	NR
50	2024	Fetal-liver MSC vs adult BM-MSC	Yes	Yes	NR	No/omics-focused	NR	Yes
51	2025	iPSC-derived MSC vs AD-MSC	Yes	NR	NR	Yes	Yes	NR
52	2025	BM-MSC vs AD-MSC vs umbilical MSC	Yes	NR	NR	Yes	Yes	NR
53	2025	Glioblastoma vs neuron/astrocyte/brain endothelial cell	Yes	Yes	NR	Yes	Yes	Yes
54	2025	BM-MSC vs cardiomyocyte	Yes	NR	NR	No/omics-focused	Yes	NR
55	2025	iPSC vs AD-MSC vs BM-MSC	Yes	Yes	NR	No/omics-focused	NR	Yes
56	2025	Induced MSC vs BM-MSC	Yes	NR	NR	Yes	NR	NR
57	2025	AD-MSC vs UC-MSC	Yes	NR	NR	No/omics-focused	Yes	NR
58	2025	Placental, endometrial and dental-pulp MSC types	Yes	Yes	NR	No/omics-focused	Yes	Yes
59	2025	iPSC-cardiomyocyte vs MSC	Yes	NR	NR	No/omics-focused	NR	Yes
60	2025	Neural progenitor vs developing neuron	Yes	NR	NR	No/omics-focused	NR	NR
61	2025	Tenocyte vs MSC exosomes	Yes	NR	NR	Yes	NR	NR
62	2026	UC-MSC vs AD-MSC	Yes	NR	NR	Yes	Yes	NR
63	2026	Canine placental MSC vs AD-MSC	Yes	NR	NR	Yes	Yes	NR
64	2026	iPSC-microglia vs neural stem cell	Yes	NR	NR	No/omics-focused	NR	NR
65	2026	MSC vs NK-cell EVs	Yes	NR	NR	Yes	NR	NR
66	2026	Chondroprogenitor vs chondrocyte vs BM-MSC	Yes	NR	NR	Yes	Yes	NR
67	2026	iPSC-MSC vs UC-MSC	Yes	NR	NR	No/omics-focused	NR	NR
68	2026	BM-MSC vs AD-MSC	Yes	NR	NR	Yes	Yes	NR
69	2026	UC-MSC vs AD-MSC	Yes	NR	NR	No/omics-focused	Yes	NR
70	2026	Six source-defined cell types	Yes	NR	NR	No/omics-focused	NR	NR
